# Synthesis and Antioxidant Activity of Caffeic Acid Derivatives

**DOI:** 10.3390/molecules23092199

**Published:** 2018-08-30

**Authors:** Katarzyna Sidoryk, Anna Jaromin, Nina Filipczak, Piotr Cmoch, Marcin Cybulski

**Affiliations:** 1 Pharmaceutical Research Institute, 8 Rydygiera Street, 01-793 Warsaw, Poland; p.cmoch@ifarm.eu (P.C.); m.cybulski@ifarm.eu (M.C.); 2 Department of Lipids and Liposomes, Faculty of Biotechnology, University of Wroclaw, 14a Joliot-Curie Street, 50-383 Wroclaw, Poland; anna.jaromin@uwr.edu.pl (A.J.); nina.filipczak@uwr.edu.pl (N.F.)

**Keywords:** phenolic acids, derivatives of caffeic acid, Wittig reaction, water medium, antioxidant activity

## Abstract

A series of caffeic acid derivatives were synthesized via a modified Wittig reaction which is a very important tool in organic chemistry for the construction of unsaturated carbon–carbon bonds. All reactions were performed in water medium at 90 °C. The aqueous Wittig reaction worked best when one unprotected hydroxyl group was present in the phenyl ring. The olefinations in the aqueous conditions were also conducted with good yields in the presence of two unprotected hydroxyl groups. When the number of the hydroxyl groups was increased to three, the reaction yields were worse, and the derivatives **12**, **13**, and **18** were obtained with 74%, 37%, and 70% yields, respectively. Nevertheless, the Wittig reaction using water as the essential medium is an elegant one-pot synthesis and a greener method, which can be a safe alternative for implementation in organic chemistry. The obtained compounds were tested for their antioxidant activity, and **12**, **13**, and **18** showed the highest activities. Moreover, all synthesized compounds displayed no cytotoxicity, and can therefore be used in the pharmaceutical or cosmetic industry.

## 1. Introduction

Caffeic acid (CA) is a natural phenolic acid which is synthesized by plants as a secondary metabolite. CA and its natural and synthetic derivatives show potent antioxidant activity, even in low concentrations. Moreover, it has been proved in many biological investigations that caffeic acid and its analogues also display anti-inflammatory, antibacterial, antiviral, and antitumor activities [1,2,3,4,5]. Recent investigations have demonstrated that caffeate esters, especially methyl caffeate, display sucrase and maltase inhibition [6,7,8].

Various biological studies on the phenolic acid molecules which have been carried out over the years have revealed that their varied biological activities stem from their antioxidant properties [9,10]. The antioxidant activity of phenolic acids follows from their acclaimed capability to scavenge reactive oxygen species (ROS) which include radical and nonradical oxygen species, such as O_2_^−^, HO, NO, and H_2_O_2_ [11,12,13]. The latest studies have disclosed that the presence of free phenolic hydroxyl groups and their number and position in the phenyl ring are essential for the strength of the antioxidant activity, while their protection renders them inactive [14]. Furthermore, the type of spacer between the carboxylic group and the aromatic ring of the phenolic acid also markedly influences their antioxidant profile—derivatives with methylenic, ethylenic, and unsaturated chains display the highest activity [15,16].

Due to advantageous biological effects, phenolic acids and their derivatives have become an essential instrument for the prevention or treatment of many diseases, and they have therefore found wide application in the cosmetic and pharmaceutical industries [17,18]. Phenolic acids, most notably caffeic acid, are also important scaffolds for the synthesis of a variety of biologically active compounds [19].

**Scheme 1:**
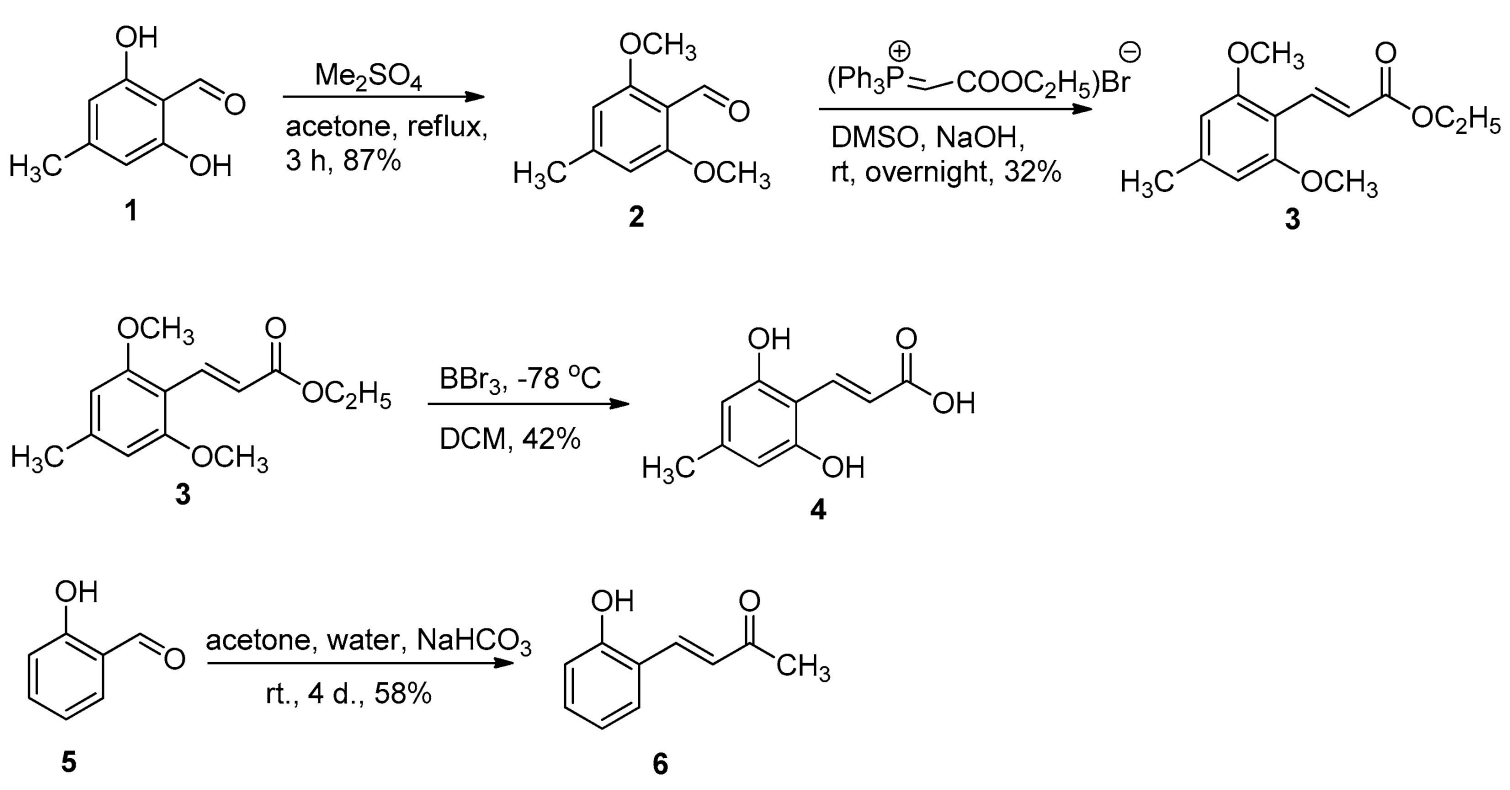
Examples of caffeic acid analogues’ preparation.

Simple commercially available phenolic acids are usually obtained by means of their extraction from natural plant sources, as well as by a biotechnological process [20]; however, chemical synthesis also plays an important role in accessing pure phenolic acids. In general, there are two most important routes to obtaining α, β-unsaturated systems [21,22,23,24]. The first approach uses a modified Wittig reaction, namely the Horner–Wadsworth–Emmons (HWE) reaction. The second exploits Knoevenagel and aldol condensations. Unfortunately, the straightforward application of these known methods to obtain different analogues of caffeic acid has drawbacks, such as the multiplicity of procedures, side reactions, high temperature, long reaction time, low yield, and low stereoselectivity (see Scheme 1) [7,14,25]. For example, the reference method of **4** synthesis yields only 12%, and the synthetic protocol contains the problematic deprotection of **3** by its demethylation at −78 °C in the presence of aggressive boron tribromide. Besides this, an alternative procedure to the modified Wittig olefination, based on the aldol condensation of aldehyde **5** with acetone, requires a prolonged reaction time, up to four days, to afford **6** with a moderate 58% yield.

**Scheme 2:**
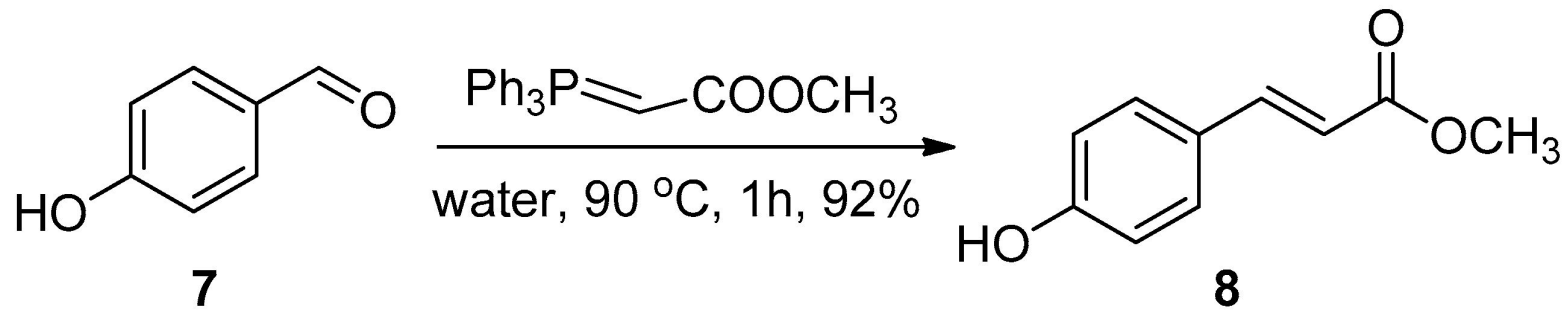
A Wittig reaction of *p*-benzaldehyde with ylide in water.

To improve the HWE reaction, a number of variations on the reaction conditions have been reported. These optimizations include, e.g., increasing the temperature or pressure, the presence of additives, irradiation with microwaves or light, and use of silica gel or ionic solvents [26,27,28,29,30]. The latest investigation demonstrated that the HWE reaction could be conducted in water, which appeared to be an efficient medium, particularly for aromatic aldehydes [31]. Although the starting material and reaction products were very poorly soluble in water, the rate of reactions was fast. Moreover, the high yields of 80–98%, short olefinations time, and very high *E*/*Z*-isomeric ratios in the olefination products prompted a detailed study of water as the essential medium in modified Wittig reactions [21,31,32]. While investigating the HWE reaction, the Bergdahl group demonstrated in one experiment that the water medium can be very effective for the *p*-hydroxybenzaldehyde substrate, having an unprotected phenolic group (Scheme 2) [31,32]. The appropriate cinnamate product **8** was obtained with a 92% yield, contrary to the same product in refluxing dichloromethane (DCM) which provided **8** with only an 8% yield [33].

This literature example encouraged us to study the HWE reaction in water using various aromatic aldehydes containing one or more unprotected hydroxyl groups in order to obtain a group of caffeic acid derivatives for biological evaluation. Moreover, the prospect of developing a synthetic method in water as the medium, to obtain the designed structures, seemed to be attractive because of mild reaction conditions and the ease of manipulation. The most important advantage of the investigated procedure, in comparison to the methods with a protected hydroxyl group, was the elimination of environmentally unsafe reagents (e.g., BBr_3_) and harsh reaction conditions.

Herein, we report a broad application of the HWE reaction in water to obtain different caffeate derivatives. Various aromatic aldehydes with the hydroxyl group in diverse positions of the aromatic ring (*p*-, *m*-, *o*-benzaldehydes) or with additional hydroxyl groups (two or three hydroxyl groups in the aromatic ring), as well as different ylides, were verified as reaction substrates. Finally, the synthesized caffeic acid derivatives were tested for their antioxidant activity and cytotoxic effects on normal human dermal fibroblasts.

## 2. Results and Discussion

### 2.1. Chemistry

Inspired by the Bergdahl reports [31,32], we have investigated different aromatic aldehydes with unprotected hydroxyl groups (Table 1) in HWE reaction conditions. Although all the starting materials and reaction products were poorly soluble in water, the reaction yields were generally high. The presence of only one hydroxyl unprotected group in the substrate aromatic ring resulted in transformations ranging from 82 to 97% (Table 1, entries 1, 2, 3, 7 and 8, 9, 10, 14). However, for the Ph_3_PCHCOCH_3_ ylide substrate, a longer time was required to complete the reaction (Table 1, entries 8, 9, and 10). The *E* isomers were isolated as the major products.

The reaction of aromatic aldehydes with two unprotected hydroxyl groups in the aromatic ring resulted in favorable yields. Derivatives **11** and **17** were achieved with yields of 86% and 78%, respectively. In contrast, the modified Wittig protocol worked very well for 4′-hydroxy-3-metoxybenzaldehyde (Table 1, entries 7 and 14). The reactions with both ylides **I** and **II** in water for 30 min at 90 °C led to **14** and **20** with 99% and 98% yields, respectively.

The introduction of a third hydroxyl group to the aromatic ring led to poorer results (Table 1, entries 5, 6, 12, and 13). Compounds **12** and **18** were obtained with moderate 74% and 70% yields, respectively, in 1–0.5 h at 90 °C, while the prolonged reaction time caused the decomposition of the substrates. Another cinnamate product **13** was obtained with just a 37% yield. This resulted from the difficulties in isolating and purifying **13** from a much more impure crude material. The reaction of 2,4,5-trihydroxybenzaldehyde with the Ph_3_PCHCOCH_3_ ylide in water did not give product **19**, even though the reaction time was extended to 5 h. Generally, a greater number of hydroxyl electron-donating groups in the benzaldehyde aromatic ring decreased the effectiveness of the conversion. This is consistent with the reported findings stating that electron-donating groups reduce the rate of the HWE reaction in methanol [31,32].

The structures of all compounds were confirmed by a detailed ^1^H and ^13^C-NMR analysis (Section 3 and Supplementary Materials), as well as by HRMS experiments. In all examples the modified Wittig conditions generated the *E*-product that corresponded to the reference observation [31,32]. Our study proved that water was an effective medium for the HWE reaction. The one-step procedure provided products with high selectivity and good yields and its application for the synthesis of different phenolic compounds turned out to be easy, fast, and environmentally friendly.

### 2.2. Biological Activity

#### 2.2.1. Antioxidant Activity of CA Derivatives

The antioxidant effect of CA and its derivatives was tested against lipid peroxidation in the o/w emulsion model (Table 2). The high antioxidant activity of **12**, **13**, and **18** manifested in the highest protection against the induced oxidation results from the presence of three hydroxyl groups causing its low lipophilicity as suggested by calculated log *P*_o/w_ values. These results are generally in agreement with the radical scavenging activities determined by using a DPPH method. The DPPH assay relies on the measurement of discoloration, the effect of the reduction of a DPPH free radical caused by an antioxidant. Thus, lower IC_50_ values for **12** and **18** from the DPPH test express their high antioxidant activities. However, it seems that the presence of one −OH group is not sufficient to exert such an activity and **9**, **10**, **8**, **6**, **15**, and **16** have no scavenging ability.

#### 2.2.2. Effects of CA Derivatives on NHDF Cell Viability

Having proven the potent antioxidant activity of the synthesized compounds and in the light of encouraging reports concerning significant protection against UV-radiation-induced skin damage [34], reduction of UV-induced skin aging [35], and anti-wrinkling activity [36], we went on to determine their preliminary cytotoxicity against skin cells.

**Figure 1:**
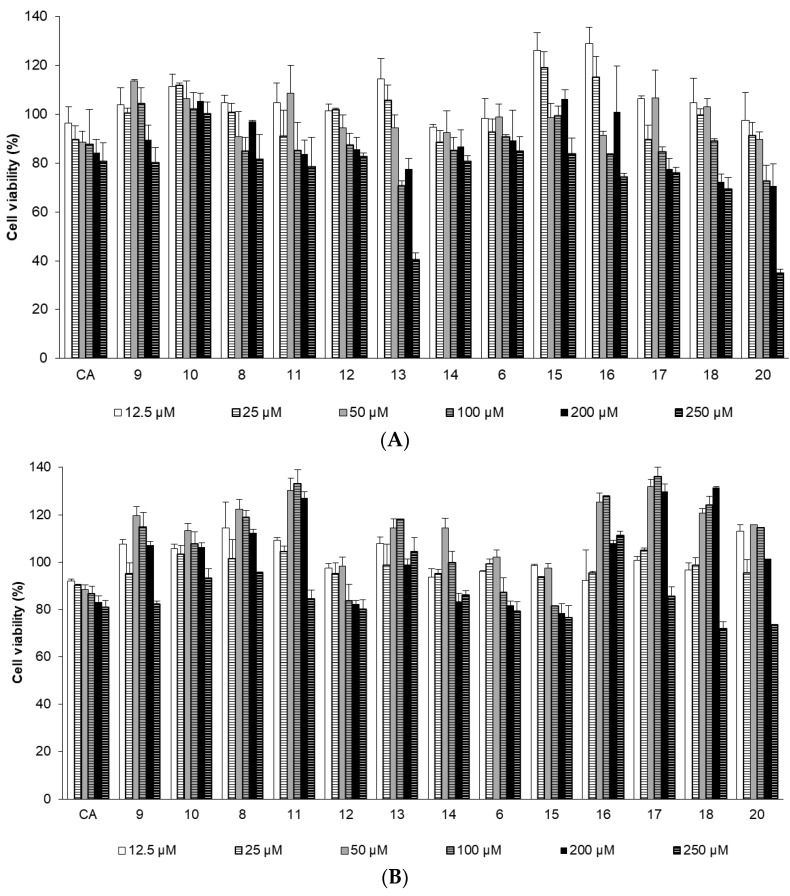
Cell viability in normal human dermal fibroblast (NHDF) cells after 24 h (**A**) and 48 h (**B**) exposure to different concentrations of CA and its derivatives. The cell viability was evaluated with MTT assay and the results were presented as the percentage of the control groups. The data are presented as the mean standard deviation (SD) of three independent experiments.

In this study we examined the biocompatibility of the synthesized compounds on the normal human dermal fibroblast (NHDF) cell line by MTT assay. Figure 1A,B show the viability of NHDF incubated with compounds at concentrations of 12.5–250 μM. All compounds did not affect the viability of human cells in vitro for 24 and 48 h, except for **13** and **20** at the highest concentration (24 h). However, in the case of these two compounds, this effect after 48 h was not clearly noticeable, probably due to their selective pumping out. This observation is very interesting taking into account the potential use of these active agents as components of skin or hair care products. However, in order to confirm their cosmeceutical potential and establish their bioavailability after topical application, several additional evaluations are needed, such as skin irritation and sensitization tests, development of optimal formulations, and evaluation of their effectiveness, as well as skin permeation studies.

## 3. Experimental

### 3.1. Chemistry

#### 3.1.1. General Materials and Methods

All reagent and solvents were purchased from common commercial suppliers without further purification. For monitoring the reaction progress, Merck DC-Alufolien Kieselgel 60 F_25_ TLC plates were used. The column chromatography was performed on Merck silica gel 60 230–400 mesh (Merck Group, Darmstadt, Germany). Melting points were measured on a Mettler Toledo MP90 apparatus (Mettler-Toledo GmbH, Greifensee, Switzerland) and were uncorrected. NMR spectra were recorded on Varian VNMRS 500 and Varian VNMRS 600 (Varian Medical Systems Inc., Palo Alto, CA, USA) spectrometers (at 298 K) in CDCl_3_ or CD_3_OD using TMS as an internal standard. The ESI-MS spectra were recorded on a PE Biosystems Mariner mass spectrometer (Thermo Fisher Scientific, Waltham, MA, USA).

#### 3.1.2. General Method for the HWE Reaction in Water

A suspension of an appropriate aromatic aldehyde (1 eq.), and ylide **I** or **II** (1.3–1.5 eq.) in water (4–10 mL) was stirred at 90 °C for 0.5–24 h. Next, the heterogeneous reaction mixture was cooled to room temperature, and the aqueous phase was extracted with DCM (3 × 10 mL). The solvent was evaporated under diminished pressure. Column chromatography (hexane–ethyl acetate, or chloroform–methanol) of the residue gave the *E*-alkene as the major product.

*3-(2.′-Hydroxyphenyl)-(E)-propenoic acid methyl ester (***9***)*. **9** was obtained from 2-hydroxybenzaldehyde (0.82 mmol, 85 μL) and (methoxycarbonylmethyl)triphenylphosphine (1.23 mmol, 410 mg). Reaction was in water (4.0 mL) at 90 °C for 0.5 h. The crude product **9** was purified by column chromatography (EtOAc/hexane, 10:1 → 3:1) to give 82% (120 mg) of **9** as a white solid. mp. 118–119 °C (lit. mp.: 119–120 °C [37]); NMR consistent with literature data [37]; HR-MS (ES) calcd. for C_10_H_10_O_3_ (M)^−^: 177.0552. Found: 177.0551.

*3-(3′-Hydroxyphenyl)-(E)-propenoic acid methyl ester (***10***).***10** was obtained from 3-hydroxybenzaldehyde (0.82 mmol, 100 mg) and (methoxycarbonylmethyl)triphenylphosphine (1.23 mmol, 410 mg). Reaction was in water (4.0 mL) at 90 °C for 1 h. The crude product **10** was purified by column chromatography (EtOAc/hexane, 10:1 → 3:1) to give 86% (126 mg) of **10** as a white solid. mp. 78–81 °C (lit. mp.: 79–81 °C [37]); NMR consistent with literature data [38]; HR-MS (ES) calcd. for C_10_H_10_O_3_ (M)^−^: 177.0552. Found: 177.0555.

*3-(4′-Hydroxyphenyl)-(E)-propenoic acid methyl ester (***8***).***8** was obtained from 4-hydroxybenzaldehyde (0.65 mmol, 80 mg) and (methoxycarbonylmethyl)triphenylphosphine (0.98 mmol, 328 mg). Reaction was in water (4.0 mL) at 90 °C for 1 h. The crude product **8** was purified by column chromatography (EtOAc/hexane, 10:1 → 3:1) to give 91% (106 mg) of **8** as a white solid. mp. 132–134 °C (Lit. mp. 128–133 °C [32]); NMR consistent with literature data [33]; HR-MS (ES) calcd. for C_10_H_10_O_3_ (M)^−^: 177.0552. Found: 177.0552.

*3-(2′,4′-dihydroxyphenyl)-(E)-propenoic acid methyl ester* (**11**). **11** was obtained from 2,4-dihydroxybenzaldehyde (0.72 mmol, 100 mg) and (methoxycarbonylmethyl)triphenylphosphine (1.08 mmol, 363 mg). Reaction was in water (5.0 mL) at 90 °C for 5 h. The crude product **11** was purified by column chromatography (EtOAc/hexane, 1:1) to give 86% (120 mg) of **11** as a white solid. mp. 158–160 °C. ^1^H-NMR (500 MHz, CDCl_3_ + CD_3_OD) δ 7.93 (d, 1H, C*H*^β^ = CH^α^, *J* = 16 Hz), 7.31 (d, aromatic, 1H, *J* = 8.5 Hz), 6.44 (d, 1H, CH^β^ = C*H*^α^, *J* = 16 Hz), 6.35 (dd, aromatic, 1H, *J* = 2 Hz, *J* = 8.5 Hz), 6.31 (d, aromatic, 1H, *J* = 2 Hz), 3.77 (s, CH_3_, 3H); ^13^C-NMR (125 MHz, CDCl_3_ + CD_3_OD) δ 169.2, 160.1, 158.1, 141.2, 130.4, 113.8, 113.5, 107.7, 102.4, 51.2; HR-MS (ES) calcd. for C_10_H_10_O_4_ (M)^−^: 193.0501. Found: 193.0503.

*3-(2′,3′,4′-trihydroxyphenyl)-(E)-propenoic acid methyl ester (***12***)*. **12** was obtained from 2,3,4-trihydroxybenzaldehyde (12.1 mmol, 1.86 g) and (methoxycarbonylmethyl)triphenylphosphine (15.73 mmol, 5.26 g). Reaction was in water (65 mL) at 90 °C for 1 h. The crude product **12** was purified by column chromatography (chloroform/methanol, 10:1 → 11:1) to give 74% (1.85 g) of **12** as a bright brown solid. mp. 149–153 °C. ^1^H-NMR (500 MHz, CDCl_3_) δ 7.87 (d, 1H, C*H*^β^ = CH^α^, *J* = 16.1 Hz), 6.88 (d, aromatic, 1H, *J* = 8.5 Hz), 6.43 (d, 1H, CH^β^ = C*H*^α^, *J* = 16.1 Hz), 6.35 (d, aromatic, 1H, *J* = 8.5 Hz), 3.74 (s, CH_3_, 3H); ^13^C-NMR (125 MHz, CDCl_3_) δ 170.6, 149.6, 148.2, 143.1, 134.0, 121.2, 115.4, 114.5, 108.5, 51.8; HR-MS (ES) calcd. for C_10_H_10_O_5_ (M)^−^: 209.0450. Found: 209.0446.

*3-(2′,4′,5′-trihydroxyphenyl)-(E)-propenoic acid methyl ester (***13***).***13** was obtained from 2,4,5-trihydroxybenzaldehyde (1.29 mmol, 200 mg) and (methoxycarbonylmethyl)triphenylphosphine (1.68 mmol, 560 mg). Reaction was in water (7 mL) at 90 °C for 60 min. The crude product **13** was purified by column chromatography (chloroform/methanol, 10:1 → 1:1) to give 37% (100 mg) of **13** as a bright brown solid. mp. 150–152 °C. ^1^H-NMR (600 MHz, CD_3_OD) δ 7.91 (d, 1H, C*H*^β^ = CH^α^, *J* = 16.2 Hz), 6.89 (s, aromatic, 1H), 6.34 (s, aromatic, 1H), 6.28 (d, 1H, CH^β^ = C*H*^α^, *J* = 16.2 Hz), 3.74 (s, CH_3_, 3H); ^13^C-NMR (150 MHz, CD_3_OD) δ 170.6, 152.9, 150.8, 142.4, 139.8, 114.6, 113.7, 113.4, 104.2, 51.8; HR-MS (ES) calcd. for C_10_H_10_O_5_ (M)^−^: 209.0450. Found: 209.0447.

*3-(4′-hydroxy-3-metoxyphenyl)-(E)-propenoic acid methyl ester (***14***)*. **14** was obtained from 4-hydroxy-3-methoxybenzaldehyde (0.65 mmol, 100 mg) and (methoxycarbonylmethyl)triphenylphosphine (0.98 mmol, 329 mg). Reaction was in water (4 mL) at 90 °C for 0.5 h. The crude product **14** was purified by column chromatography (EtOAc/hexane, 5:1 → 1:1) to give 99% (134 mg) of **14** as a white solid. mp. 67–69 °C (lit. mp. 62–63 °C [16]). ^1^H-NMR (500 MHz, CDCl_3_) δ 7.63 (d, 1H, C*H*^β^ = CH^α^, *J* = 16 Hz), 7.08 (d, aromatic, 1H, *J* = 1.5 Hz), 7.07 (m, aromatic, 1H, *J* = 1.5 Hz, *J* = 2 Hz), 7.02 (m, aromatic, 1H, *J* = 1.5 Hz), 6.27 (d, 1H, CH^β^ = C*H*^α^, *J* = 16 Hz), 3.92 (s, CH_3_, 3H), 3.79 (s, CH_3_, 3H); ^13^C-NMR (125 MHz, CDCl_3_) δ 167.7, 147.9, 146.7, 144.9, 126.9, 123.0, 115.1, 114.7, 109.3, 55.9, 51.5; HR-MS (ES) calcd. for C_11_H_12_O_4_ (M)^−^: 207.0657. Found: 207.0654.

*4-(2′-hydroxyphenyl)-3(E)-buten-2-one (***6***).***6** was obtained from 2-hydroxybenzaldehyde (0.82 mmol, 85 μL) and 1-(triphenylphosphoranylidene)-2-propanone (1.23 mmol, 391 mg). Reaction was in water (4.0 mL) at 90 °C for 2.5 h. The crude product **6** was purified by column chromatography (EtOAc/hexane, 5:1 → 1:1) to give 85% (112 mg) of **6** as a yellow solid. mp. 137–139 °C (lit. mp. 139–140 °C [25]); NMR consistent with literature data [25]; HR-MS (ES) calcd. for C_10_H_10_O_2_ (M)^−^: 161.0603. Found: 161.0604.

*4-(3′-hydroxyphenyl)-3(E)-buten-2-one (***15***)*. **15** was obtained from 3-hydroxybenzaldehyde (0.82 mmol, 100 mg) and 1-(triphenylphosphoranylidene)-2-propanone (1.23 mmol, 391 mg). Reaction was in water (4.0 mL) at 90 °C for 3 h. The crude product was purified by column chromatography (EtOAc/hexane, 5:1 → 1:1) to give 97% (128 mg) of **15** as a white solid. mp. 94–96 °C (lit. mp. 96–97 °C [21]). ^1^H-NMR (500 MHz, CDCl_3_) δ 7.50 (d, 1H, C*H*^β^ = CH^α^, *J* = 16.0 Hz), 7.28–7.24 (m, aromatic, 1H), 7.10–7.08 (m, aromatic, 2H), 6.94–6.92 (m, aromatic, 1H), 6.71 (d, 1H, CH^β^ = C*H*^α^, *J* = 16.0 Hz), 2.40 (s, CH_3_, 3H); ^13^C-NMR (125 MHz, CDCl_3_) δ 199.7, 156.5, 144.2, 135.7, 130.2, 127.1, 121.0, 118.1, 114.6, 27.4; HR-MS (ES) calcd. for C_10_H_10_O_2_ (M)^−^: 161.0603. Found: 161.0602.

*4-(4′-hydroxyphenyl)-3(E)-buten-2-one (***16***)*. **16** was obtained from 4-hydroxybenzaldehyde (0.82 mmol, 100 mg) and 1-(triphenylphosphoranylidene)-2-propanone (1.23 mmol, 391 mg). Reaction was in water (4.0 mL) at 90 °C for 2 h. The crude product was purified by column chromatography (EtOAc/hexane, 5:1 → 1:1) to give 83% (110 mg) of **16** as a white solid. mp. 110–111 °C (lit. mp. 111–113 °C [38]). ^1^H-NMR (500 MHz, CDCl_3_) δ 7.53 (d, 1H, CH^β^ = CH^α^, *J* = 16.0 Hz), 7.45–7.43 (m, aromatic, 2H), 6.93–6.90 (m, aromatic, 2H), 6.62 (d, 1H, CH^β^ = C*H*^α^, *J* = 16.0 Hz), 2.39 (s, CH_3_, 3H); ^13^C-NMR (125 MHz, CDCl_3_) δ 200.1, 159.0, 144.8, 130.4, 126.4, 124.3, 116.2, 27.2; HR-MS (ES) calcd. for C_10_H_10_O_2_ (M)^−^: 161.0603. Found: 161.0609.

*4-(2′,4′-dihydroxyphenyl)-3(E)-buten-2-one (***17***)*. **17** was obtained from 2,4-dihydroxybenzaldehyde (1.44 mmol, 200 mg) and 1-(triphenylphosphoranylidene)-2-propanone (1.88 mmol, 600 mg). Reaction was in water (10.0 mL) at 90 °C for 2 h. The crude product was purified by column chromatography (EtOAc/hexane, 5:1 → 1:1) to give 78% (200 mg) of **17** as a bright brown solid; mp. 118–120 °C decomp., ^1^H-NMR (500 MHz, CD_3_OD) δ 7.91 (d, 1H, C*H*^β^ = CH^α^, *J* = 16.0 Hz), 7.39 (d, aromatic, 1H, *J* = 8 Hz), 6.71 (d, 1H, CH^β^ = C*H*^α^, *J* = 16.0 Hz), 6.34–6.32 (m, aromatic, 2H), 2.32 (s, CH_3_, 3H); ^13^C-NMR (125 MHz, CD_3_OD) δ 202.2, 162.9, 160.4, 142.6, 131.4, 124.1, 114.6, 109.1, 103.4, 26.7; HR-MS (ES) calcd. for C_10_H_10_O_3_ (M)^−^: 177.0552. Found: 177.0555.

*4-(2′,3′,4′-trihydroxyphenyl)-3(E)-buten-2-one (***18***).***18** was obtained from 2,3,4-trihydroxybenzaldehyde (1.29 mmol, 200 mg) and 1-(triphenylphosphoranylidene)-2-propanone (1.94 mmol, 620 mg). Reaction was in water (10.0 mL) at 90 °C for 0.5 h. The crude product was purified by column chromatography (EtOAc/hexane, 2:1 → 1:1) to give 70% (175 mg) of **18** as a bright brown solid; mp. 98–100 °C decomp. ^1^H-NMR (500 MHz, CD_3_OD) δ 7.88 (d, 1H, C*H*^β^ = CH^α^, *J* = 16.1 Hz), 6.96 (d, aromatic, 1H, *J* = 8.7 Hz), 6.72 (d, 1H, CH^β^ = C*H*^α^, *J* = 16.1 Hz), 6.37 (d, aromatic, 1H, *J* = 8.6 Hz), 2.33 (s, CH_3_, 3H); ^13^C-NMR (125 MHz, CD_3_OD) δ 202.1, 150.2, 148.5, 142.9, 134.0, 124.5, 121.1, 115.3, 108.7, 26.7; HR-MS (ES) calcd. for C_10_H_10_O_4_ (M)^−^: 193.0501. Found: 193.0506.

*4-(3′-methoxy-4′-hydroxyphenyl)-3(E)-buten-2-one (***20***)*. **20** was obtained from 4-hydroxy-3-methoxybenzaldehyde (0.65 mmol, 100 mg) and 1-(triphenylphosphoranylidene)-2-propanone (0.98 mmol, 313 mg). Reaction was in water (4.0 mL) at 90 °C for 0.5 h. The crude product was purified by column chromatography (EtOAc/hexane, 5:1 → 2:1) to give 98% (124 mg) of **20** as a white solid; mp. 126–128 °C (lit. mp. 126 °C [39]); ^1^H-NMR (500 MHz, CDCl_3_) δ 7.46 (d, 1H, C*H*^β^ = CH^α^, *J* = 16.0 Hz), 7.10–7.07 (m, aromatic, 1H), 7.05 (d, aromatic, 1H, *J* = 1.5 Hz), 6.92 (d, aromatic, 1H, *J* = 8 Hz), 6.57 (d, 1H, CH^β^ = C*H*^α^, *J* = 16.0 Hz), 6.03 (br s, O*H*, 1H), 3.93 (s, CH_3_, 3H), 2.36 (s, CH_3_, 3H); ^13^C-NMR (125 MHz, CDCl_3_) δ 198.4, 148.2, 146.9, 143.7, 126.8, 124.9, 123.5, 114.8, 109.3, 55.9, 27.2; HR-MS (ES) calcd. for C_11_H_12_O_3_ (M)^−^: 191.0708. Found: 191.0709.

### 3.2. Biological Activities

#### 3.2.1. Calculation of log *P*_o/w_

The partition coefficient between *n*-octanol and water (log *P*_o/w_) was calculated using the SwissADME web tool [40].

#### 3.2.2. Determination of the Oxidation Inhibition of the o/w Emulsion

The emulsion containing 15 mg of sorbitan sesquioleate, 50 mg of flaxseed oil, and 2 mL of 10 mM Tris-HCl buffer, pH 7.4, was prepared by the method [41,42]. Determination of the oxidation inhibition of the o/w emulsion was carried out according to a procedure described previously [43], using 20 μL of the 1:1 (*v*/*v*) buffer-diluted emulsion, methanolic solution of each compound (final concentration 100 μM), and ammonium iron(II) sulfate hexahydrate (Mohr′s salt) (5 mM). Thiobarbituric acid reacting substances (TBARS) were determined according to [44].

#### 3.2.3. DPPH Assay

The 2,2-diphenyl-1-picrylhydrazyl (DPPH) assay was performed using a previously reported method [45] with a modification. A series of samples consisting of 900 μL of the 0.04 mg/mL methanolic solution of DPPH and different concentrations of the compounds (100 μL) were prepared. Then, the absorbance at 517 nm was measured after a 30 min incubation at room temperature. The half maximal inhibitory concentration (IC_50_) was used as an indicating measure of the scavenging activity.

#### 3.2.4. Cell Viability Assay in the Normal Human Dermal Fibroblast (NHDF) Cell Line

The cytotoxicity test was performed as described in [44] using the NHDF cell line treated with the methanolic solution of the tested compounds to obtain the final concentrations of 12.5, 25, 50, 100, 200, or 250 µM, and incubation for 24 and 48 h. Cell viability was assessed by the MTT method [46].

## 4. Conclusions

To sum up, water is an effective medium for the HWE reaction. A one-step procedure provides *E*-products with a high selectivity and good yields and its application in the synthesis of different phenolic compounds turns out to be easy, fast, and environmentally friendly. The synthetic method for the preparation of phenolic caffeic acid derivatives in water is advantageous because of the mild reaction conditions and the ease of manipulation. The most important advantage of this procedure, in comparison to the methods with the protected phenolic groups, is the elimination of environmentally unsafe reagents (e.g., BBr_3_) and harsh reaction conditions. Moreover, the development of an efficient HWE process broadens the spectrum of the synthetic polyphenols available for potential use in the pharmaceutical or cosmetic industries. The obtained analogues of caffeic acid were tested for their antioxidant activity, and three of them (**12**, **13**, and **18**) displayed a higher antioxidant activity than caffeic acid. Exhaustive biological studies of the anti-inflammatory, antibacterial, and antiviral activity are to be the subject of further investigation.

## Figures and Tables

**Table 1:**
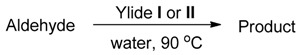
The Horner–Wadsworth–Emmons (HWE) reaction of different aromatic aldehydes in water.

Entry	Aldehyde	Ylide	Product (*E* Isomer)	Time (h)	Yield (%)
1.	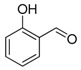	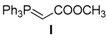	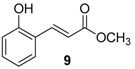	0.5	82
2.	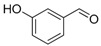	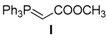	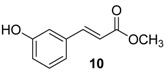	1.0	86
3.	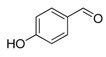	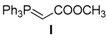	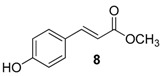	1.0	91
4.	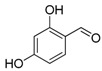	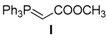	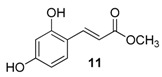	5.0	86
5.	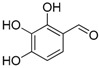	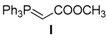	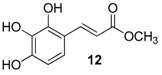	1.0	74
6.	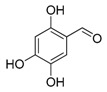	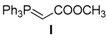	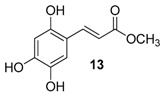	1.0	37
7.	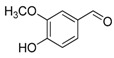	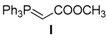	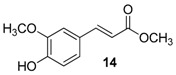	0.5	99
8.	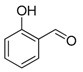	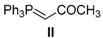	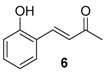	2.5	85
9.	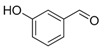	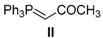	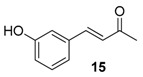	3.0	97
10.	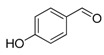	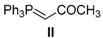	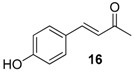	2.0	83
11.	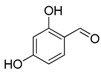	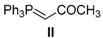	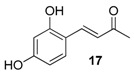	2.0	78
12.	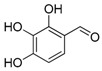	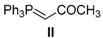	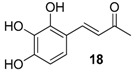	0.5	70
13.	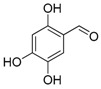	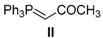	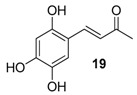	5.0	0
14.	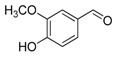	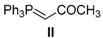	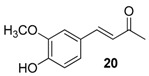	0.5	98

**Table 2:** Log *P*_o/w_, and the antioxidant activity of the tested compounds.

Compound	log *P*_o/w_^a^	Inhibition of Oxidation of o/w Emulsion ^b^	DPPH IC_50_ (µM)
CA	0.93	26.1 ± 2.9	32.2
9	1.82	2.6 ± 1.5	*n.a.* ^c^
10	1.78	18.4 ± 2.2	*n.a.* ^c^
8	1.81	22.7 ± 6.4	*n.a.* ^c^
11	1.31	33.7 ± 6.5	1015.9
12	0.99	55.5 ± 2	18.6
13	0.95	38.6 ± 2.3	53.3
14	1.76	14.8 ± 2	58.9
6	1.94	*n.a.* ^c^	*n.a.* ^d^
15	1.82	*n.a.* ^c^	*n.a.* ^d^
16	1.85	6.8 ± 4.4	*n.a.* ^d^
17	1.49	4.6 ± 1.3	688.2
18	1.05	28.6 ±4	16.8
20	1.86	7.9 ± 2.1	61.8

^a^ The prediction of log *P*_o/w_ was performed using the SwissADME program; ^b^ determined at 100 µM; ^c^ no protection detected; ^d^ IC_50_ > 5 mM. CA, caffeic acid.

## Supplementary Materials

The supplementary materials are available online.

## Abbreviations

CA	caffeic acid
HWE	Horner–Wadsworth–Emmons
DCM	dichloromethane
DPPH	2,2-diphenyl-1-picrylhydrazyl
EtOAc	ethyl acetate
DMSO	dimethyl sulfoxide
log *P*_o/w_	partition coefficient between *n*-octanol and water
MTT	3-(4,5-dimethyl-2-thiazolyl)-2,5-diphenyl-2H-tetrazolium bromide
NHDF	normal human dermal fibroblasts
TBARS	thiobarbituric acid reactive substances
