# Supplementary material for: Synthesis and Antioxidant Activity of Caffeic Acid Derivatives
Source: Molecules. 2018 Aug 30;23(9):2199. doi: 10.3390/molecules23092199 (PMC6225178; doi:10.3390/molecules23092199)
Supplement: Supplementary file 1 [file molecules-23-02199-s001.pdf]

Supporting Information

Synthesis and antioxidant activity of caffeic acid derivatives

Katarzyna Sidoryk[1], Anna Jaromin[2], Nina Filipczak[2], Piotr Cmoch[1], Marcin Cybulski[1]

[1]Pharmaceutical Research Institute, Rydygiera 8, 01-793 Warsaw, Poland

[2]Department of Lipids and Liposomes, Faculty of Biotechnology, University of Wroclaw, Joliot-Curie 14A, 50-383 Wroclaw, Poland

To whom correspondence should be addressed: Phone: +48-22-456-3928 Fax: +48-22-456-3838 E-mail: k.sidoryk@ifarm.eu

Supporting material contains ^{1}H and ^{13}C NMR spectra of compounds:

1. 3-(2'-Hydroxyphenyl)-(*E*)-propenoic acid methyl ester **(9)**
2. 3-(3'-Hydroxyphenyl)-(*E*)-propenoic acid methyl ester **(10)**
3. 3-(4'-Hydroxyphenyl)-(*E*)-propenoic acid methyl ester **(8)**
4. 3-(2',4'-dihydroxyphenyl)-(*E*)-propenoic acid methyl ester **(11)**
5. 3-(2',3',4'-trihydroxyphenyl)-(*E*)-propenoic acid methyl ester **(12)**
6. 3-(2',4',5'-trihydroxyphenyl)-(*E*)-propenoic acid methyl ester **(13)**
7. 3-(4'-hydroxy-3-metoxyphenyl)-(*E*)-propenoic acid methyl ester **(14)**
8. 4-(2'-hydroxyphenyl)-3(*E*)-buten-2-one **(6)**
9. 4-(3'-hydroxyphenyl)-3(*E*)-buten-2-one **(15)**
10. 4-(4'-hydroxyphenyl)-3(*E*)-buten-2-one **(16)**
11. 4-(2',4'-dihydroxyphenyl)-3(*E*)-buten-2-one **(17)**
12. 4-(2',3',4'-trihydroxyphenyl)-3(*E*)-buten-2-one **(18)**
13. 4-(3'-methoxy-4'-hydroxyphenyl)-3(*E*)-buten-2-one **(20)**

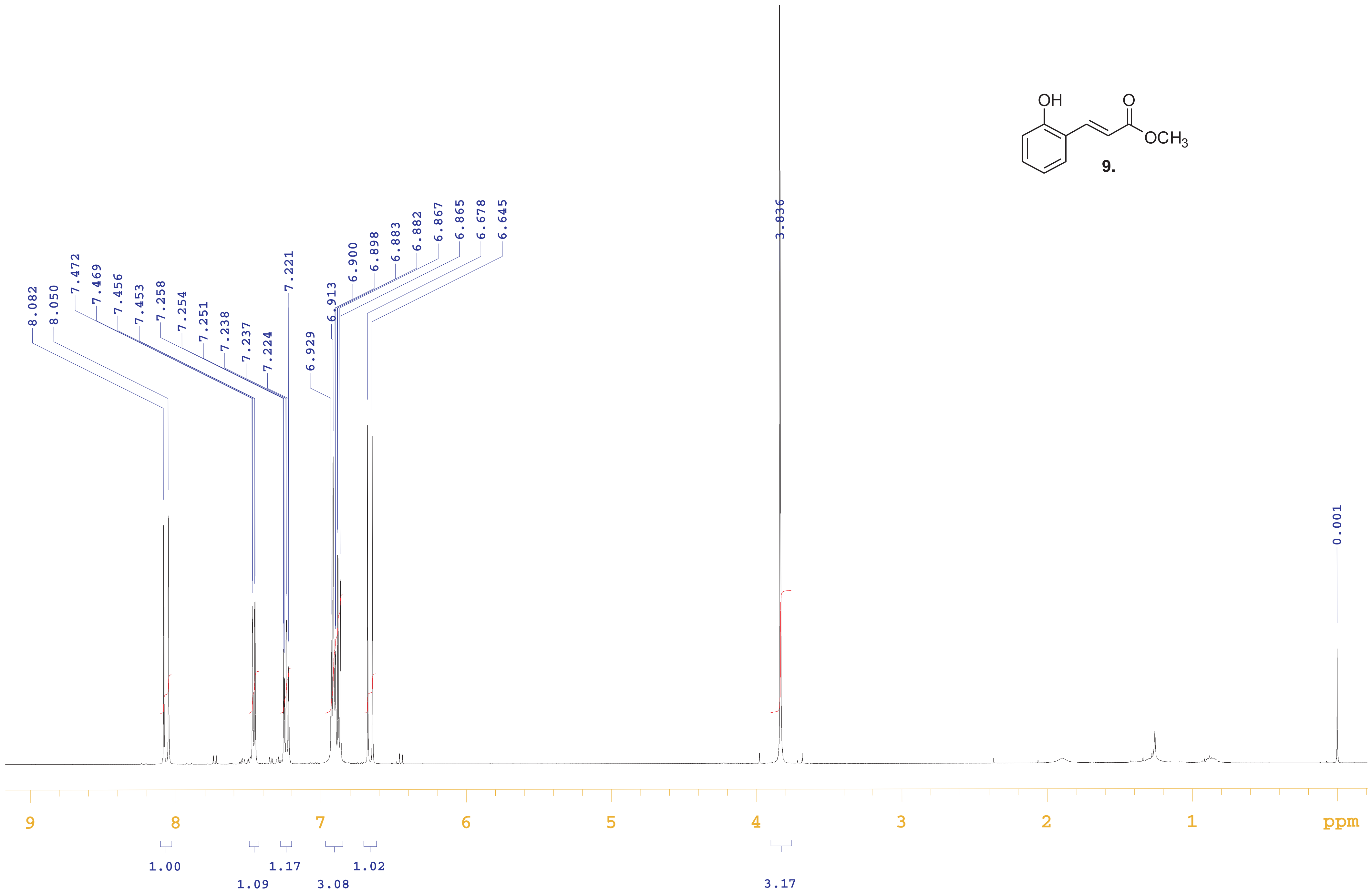
OH
O
OCH3
9.
8.082
8.050
7.472
7.469
7.456
7.453
7.258
7.254
7.251
7.238
7.237
7.224
7.221
6.929
6.913
6.900
6.898
6.883
6.882
6.867
6.865
6.678
6.645
3.836
0.001
9
8
7
6
5
4
3
2
1
ppm
1.00
1.09
1.17
3.08
1.02
3.17

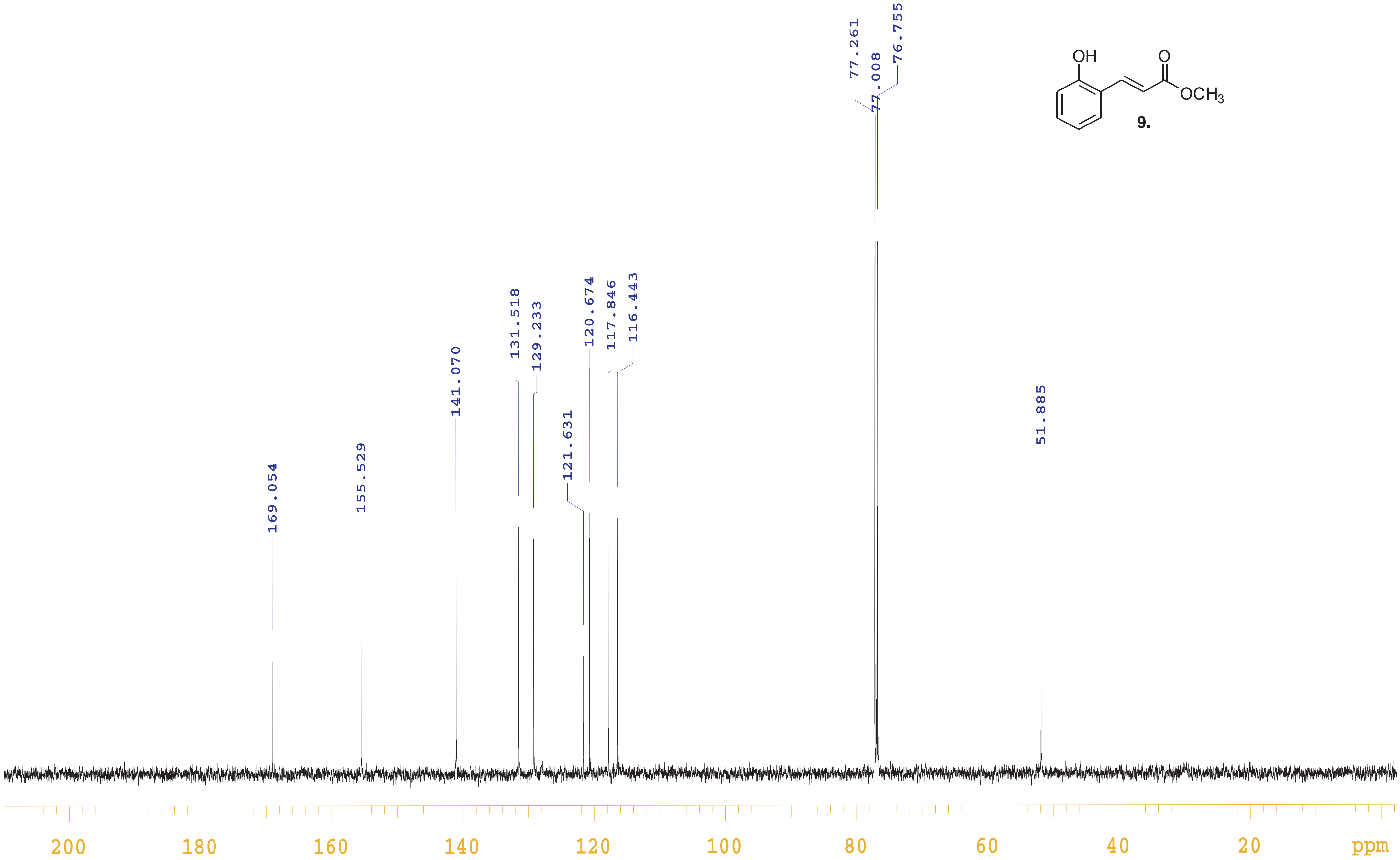

169.054
155.529
141.070
131.518
129.233
121.631
120.674
117.846
116.443
77.261
77.008
76.755
51.885
OH
O
OCH_3
9.
200
180
160
140
120
100
80
60
40
20
ppm

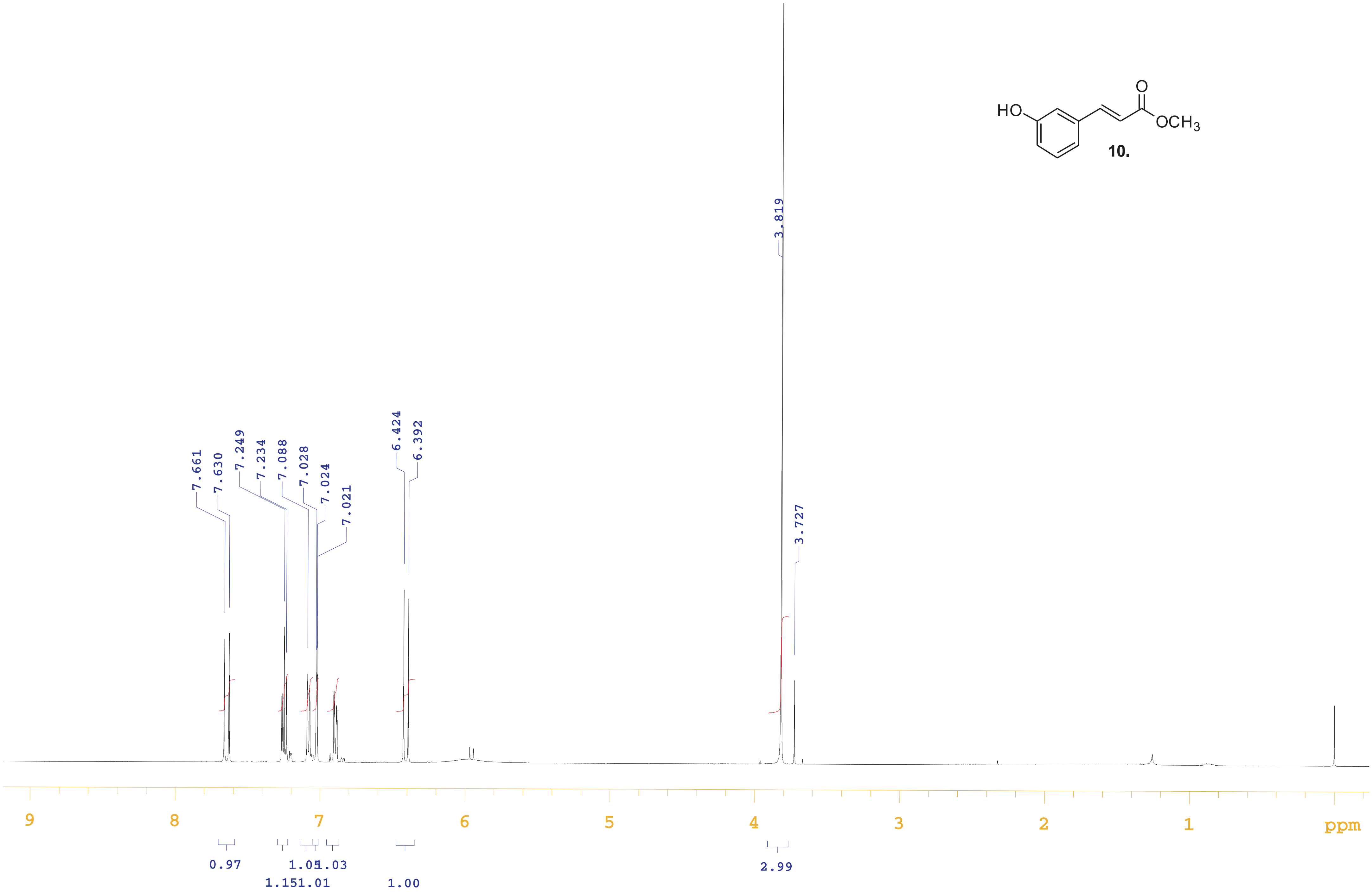
7.661
7.630
7.249
7.234
7.088
7.028
7.024
7.021
6.424
6.392
3.819
3.727
9
8
7
6
5
4
3
2
1
ppm
0.97
1.15
1.05
1.01
1.03
1.00
2.99
HO
O
OCH_3

10.

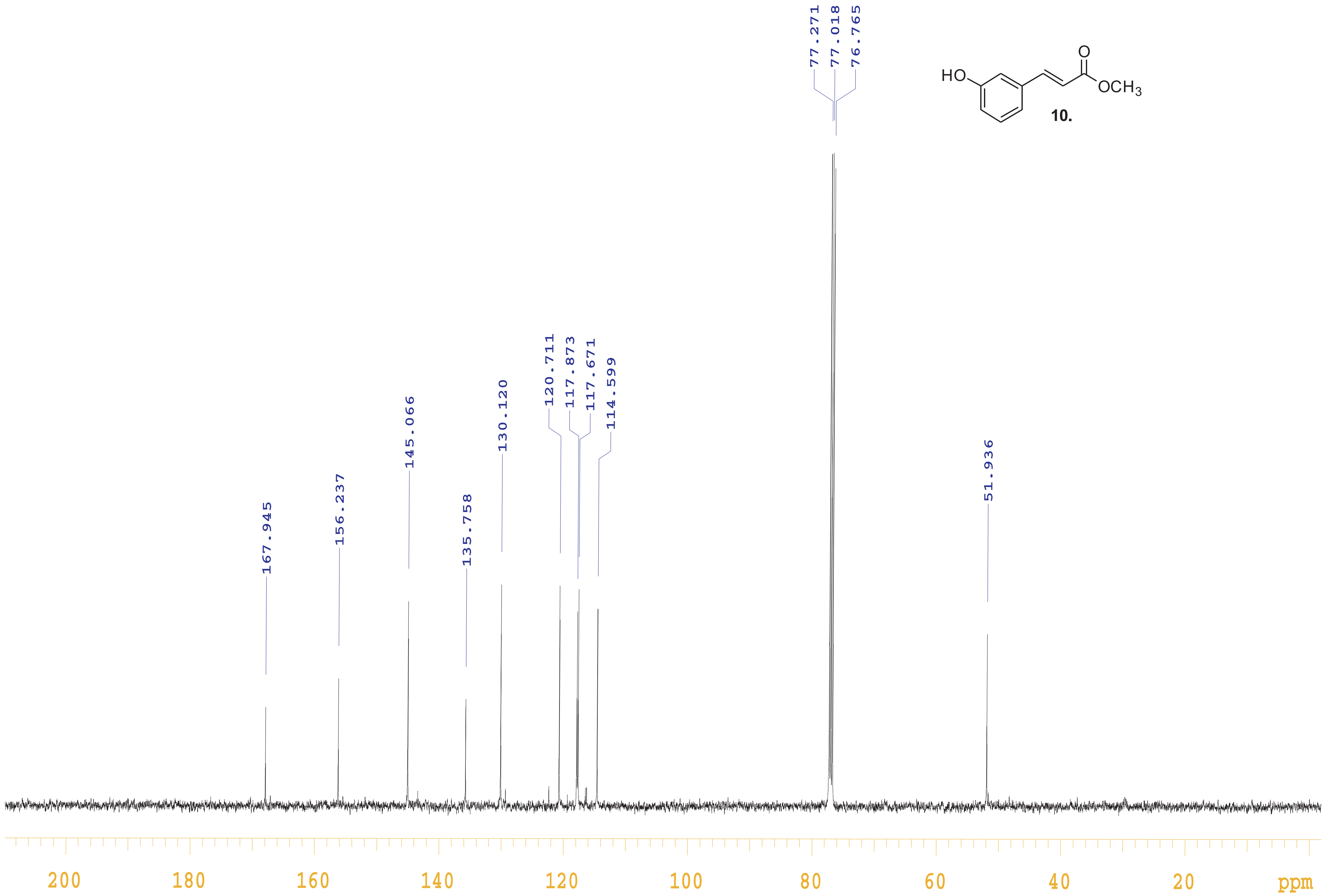

77.271
77.018
76.765
HO
O
OCH3
10.
167.945
156.237
145.066
135.758
130.120
120.711
117.873
117.671
114.599
51.936
200
180
160
140
120
100
80
60
40
20
ppm

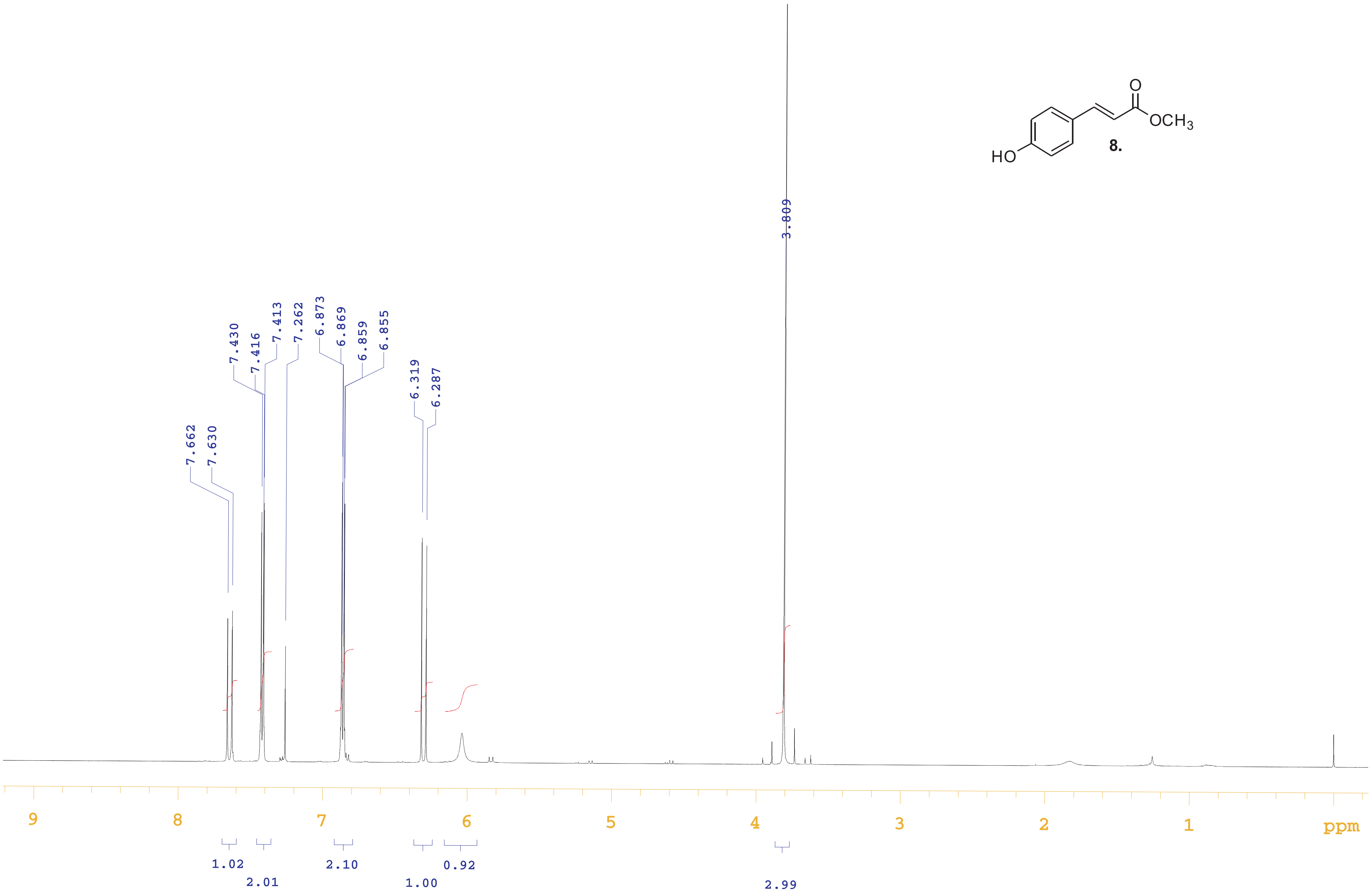

HO
O
OCH3
8.
7.662
7.630
7.430
7.416
7.413
7.262
6.873
6.869
6.859
6.855
6.319
6.287
3.809
9
8
7
6
5
4
3
2
1
ppm
1.02
2.01
2.10
1.00
0.92
2.99

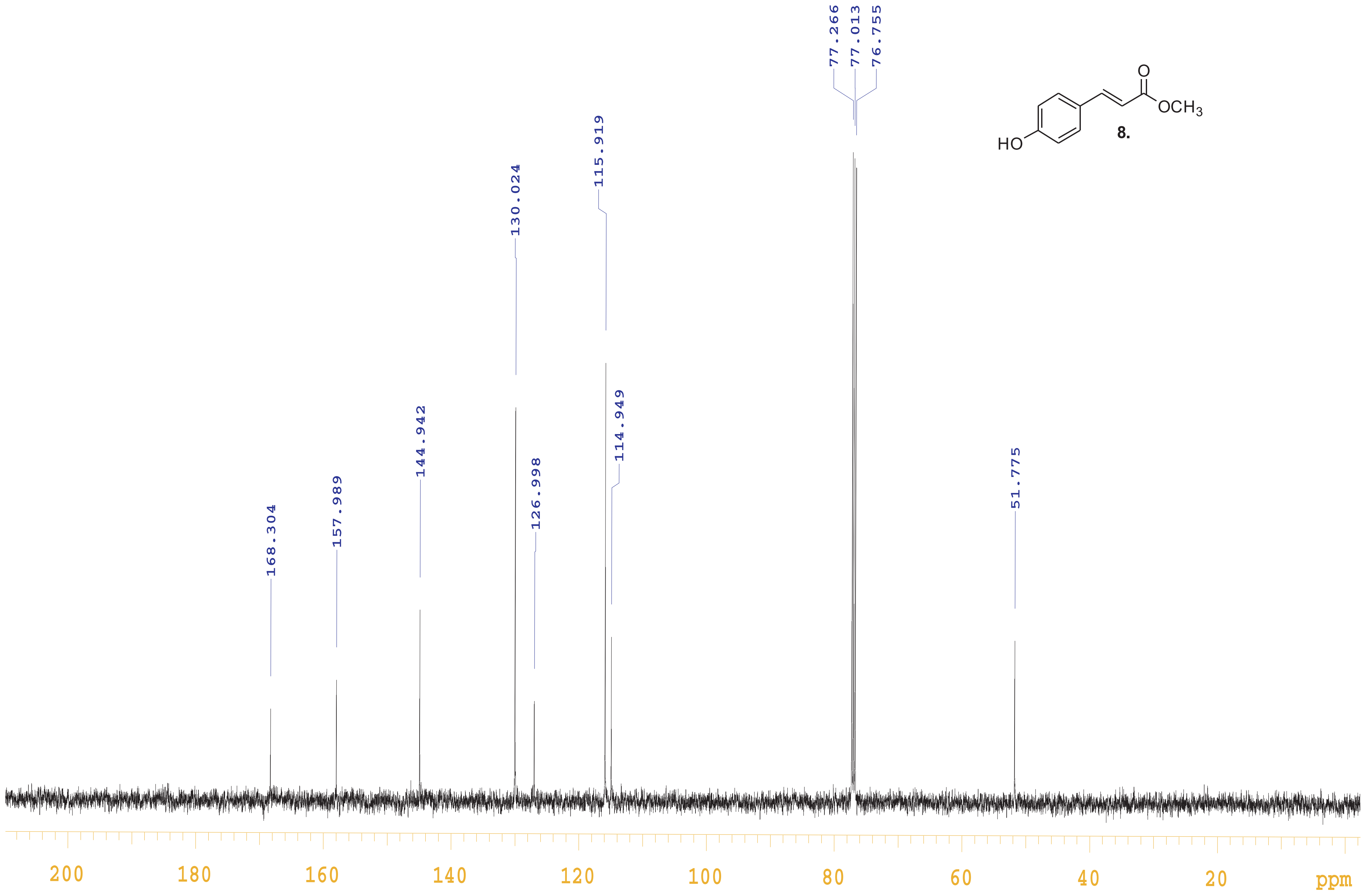

77.266
77.013
76.755
115.919
130.024
114.949
144.942
126.998
157.989
168.304
51.775
O
OCH3
HO
8.
200
180
160
140
120
100
80
60
40
20
ppm

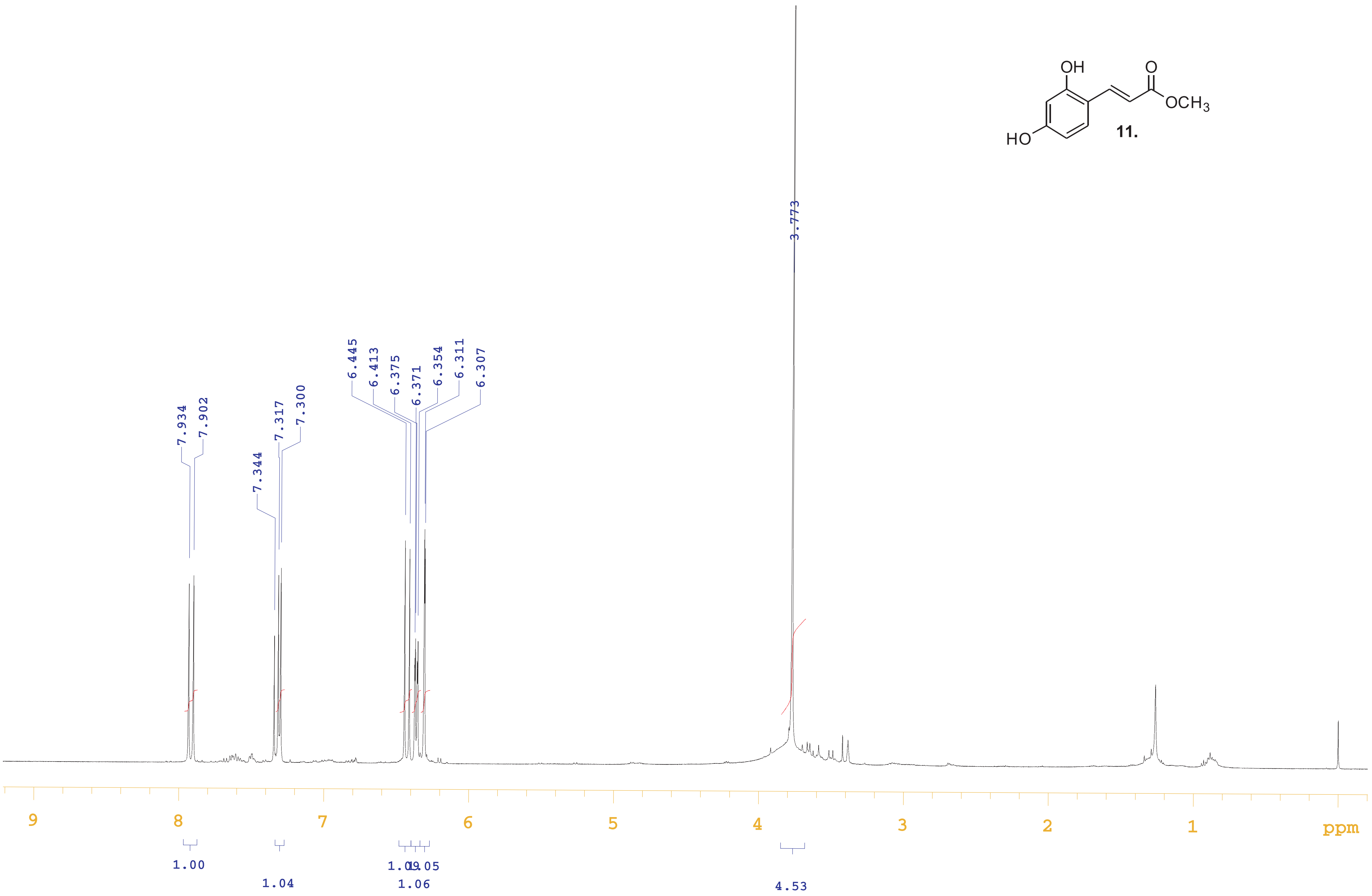
11.
OH
O
OCH3
HO
3.773
7.934
7.902
7.344
7.317
7.300
6.445
6.413
6.375
6.371
6.354
6.311
6.307
9
8
7
6
5
4
3
2
1
ppm
1.00
1.04
1.06
4.53

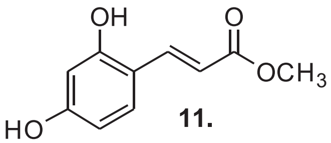
OH
O
OCH3
HO

11.

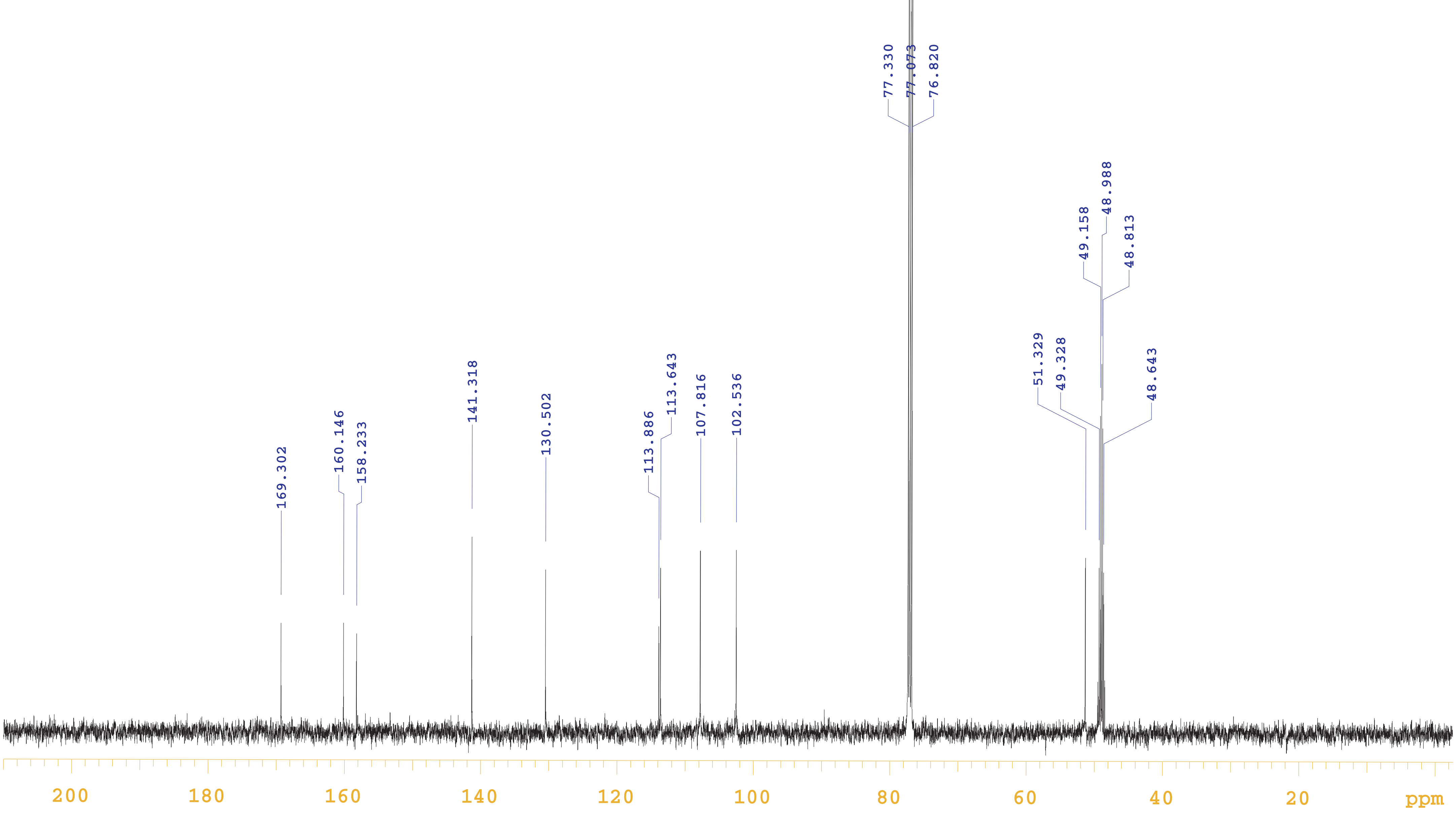
169.302
160.146
158.233
141.318
130.502
113.886
113.643
107.816
102.536
77.330
77.073
76.820
51.329
49.328
49.158
48.988
48.813
48.643
200
180
160
140
120
100
80
60
40
20
ppm

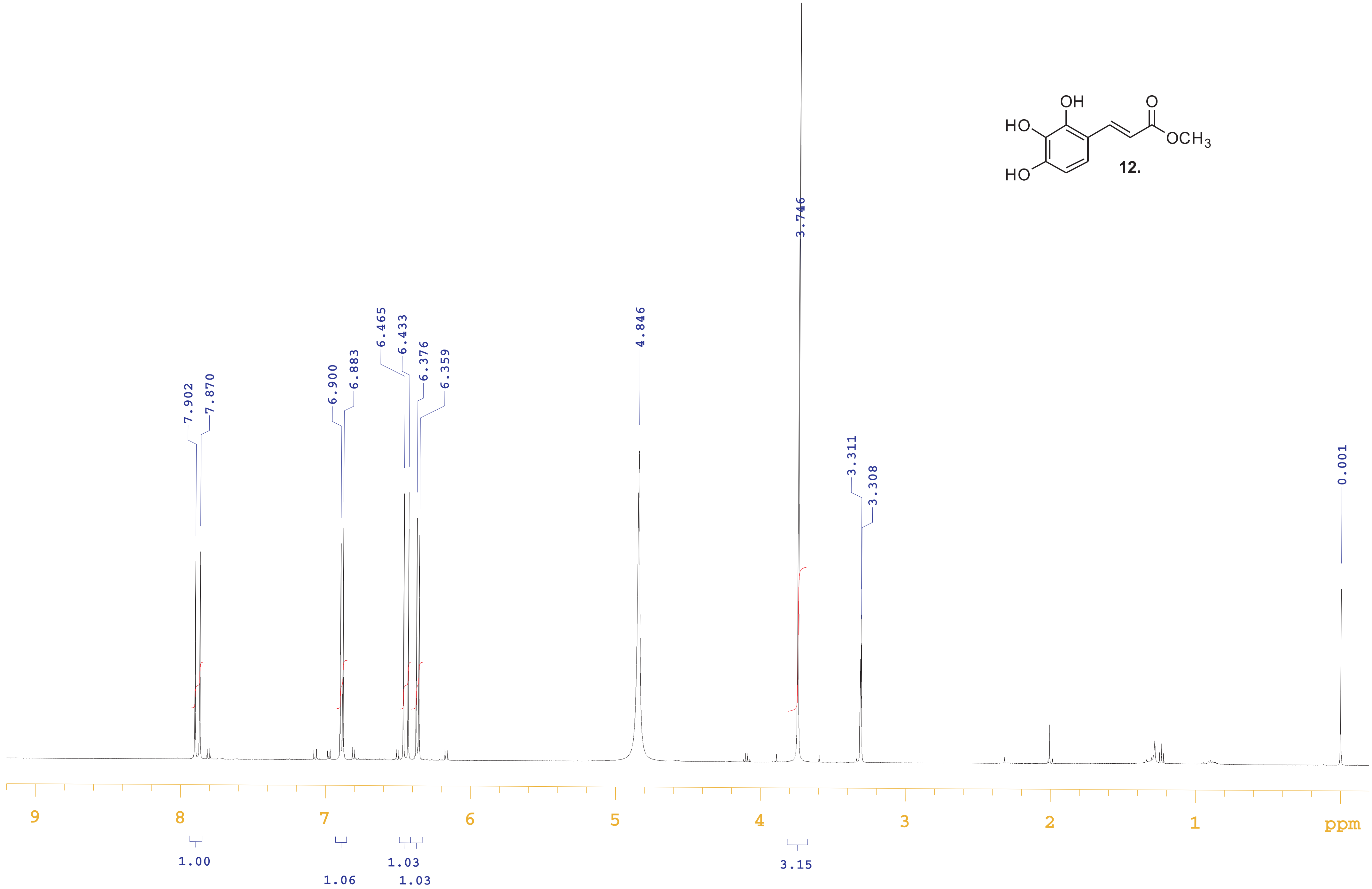

OH
HO
O
OCH3
HO
12.
7.902
7.870
6.900
6.883
6.465
6.433
6.376
6.359
4.846
3.746
3.311
3.308
0.001
9
8
7
6
5
4
3
2
1
ppm
1.00
1.06
1.03
1.03
3.15

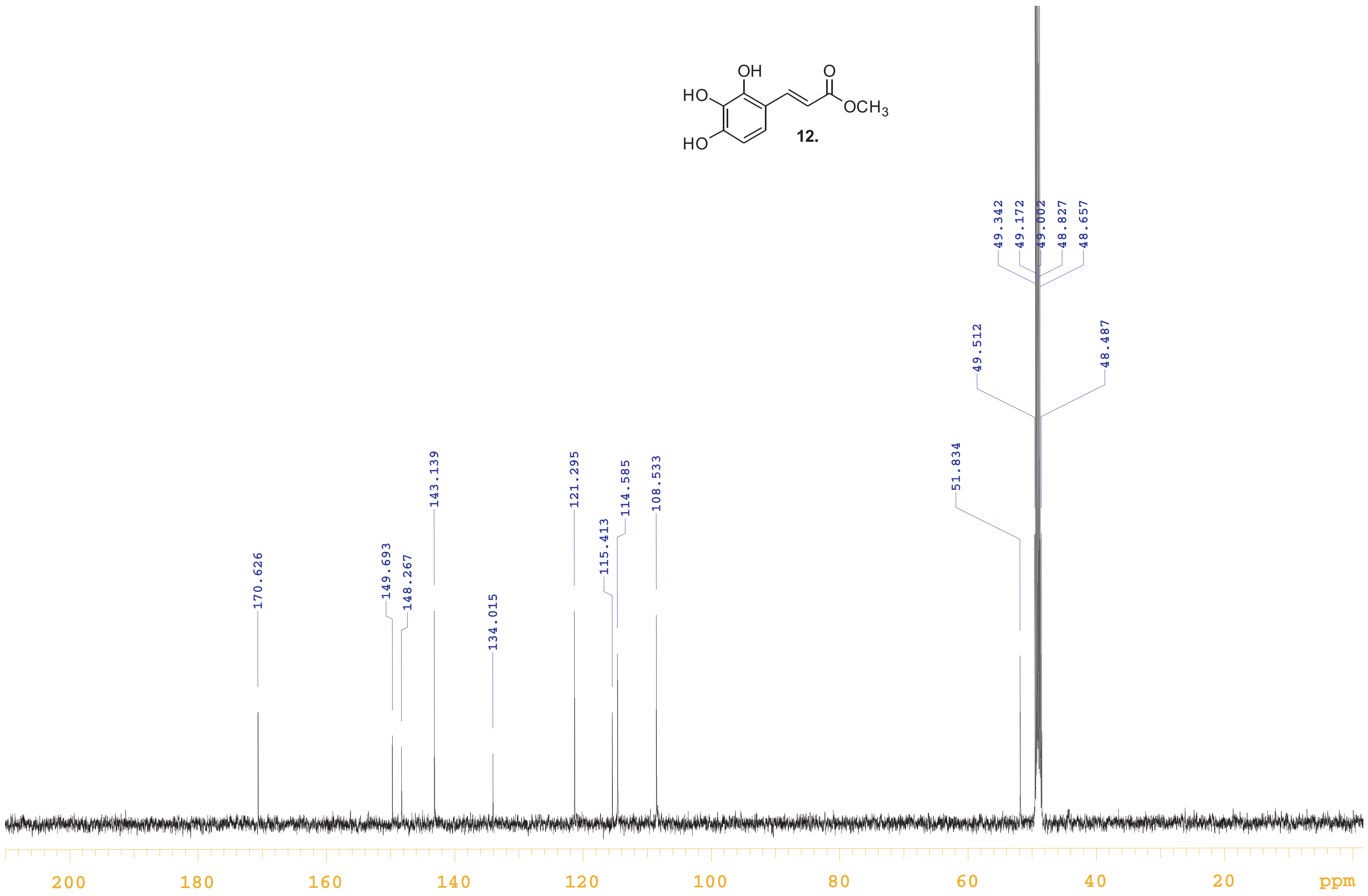
HO
OH
HO
O
OCH3
12.
49.342
49.172
49.002
48.827
48.657
49.512
48.487
51.834
170.626
149.693
148.267
143.139
134.015
121.295
115.413
114.585
108.533
200
180
160
140
120
100
80
60
40
20
ppm

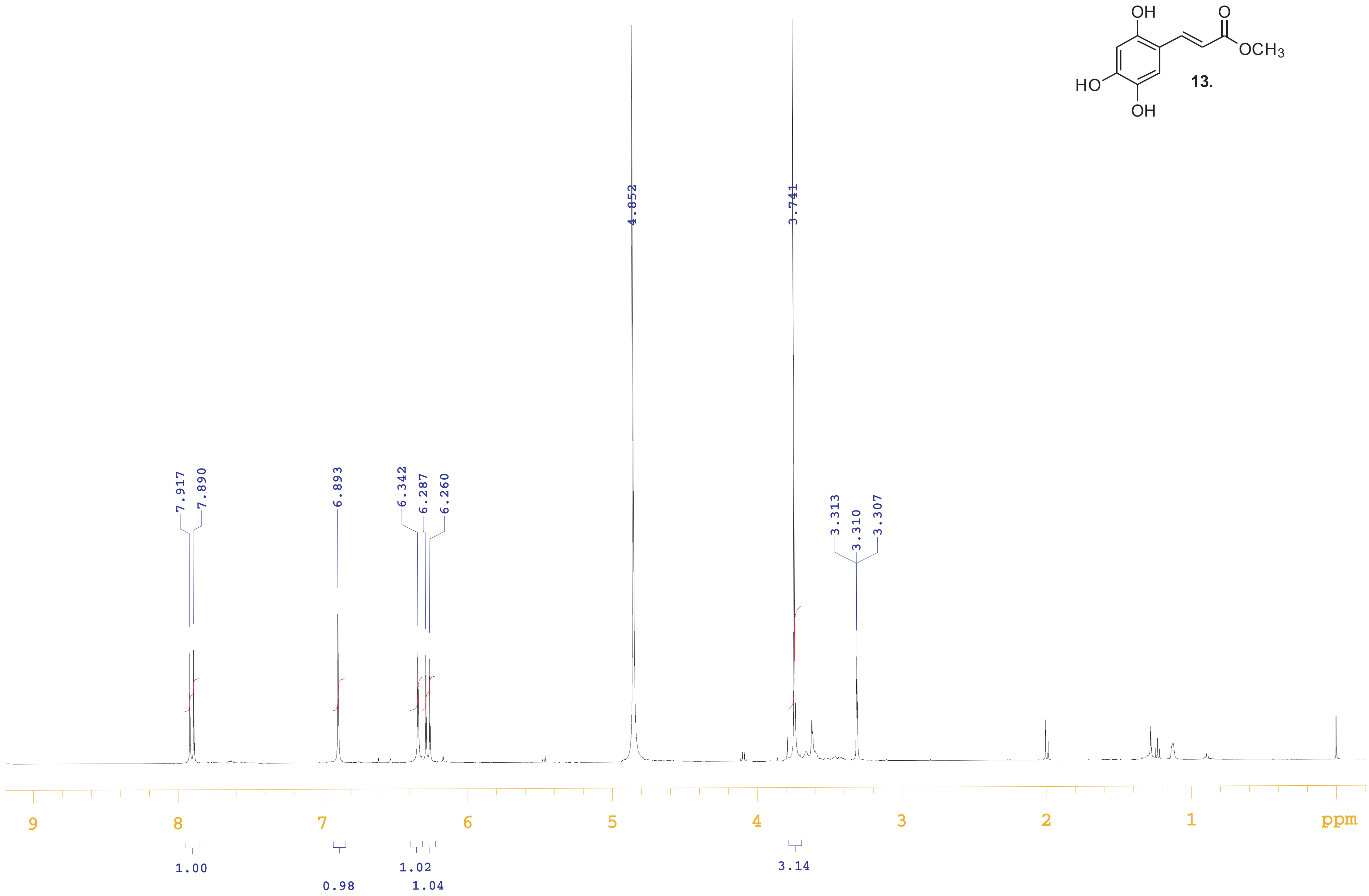
OH
O
OCH3
HO
OH
13.
7.917
7.890
6.893
6.342
6.287
6.260
4.852
3.741
3.313
3.310
3.307
9
8
7
6
5
4
3
2
1
ppm
1.00
0.98
1.02
1.04
3.14

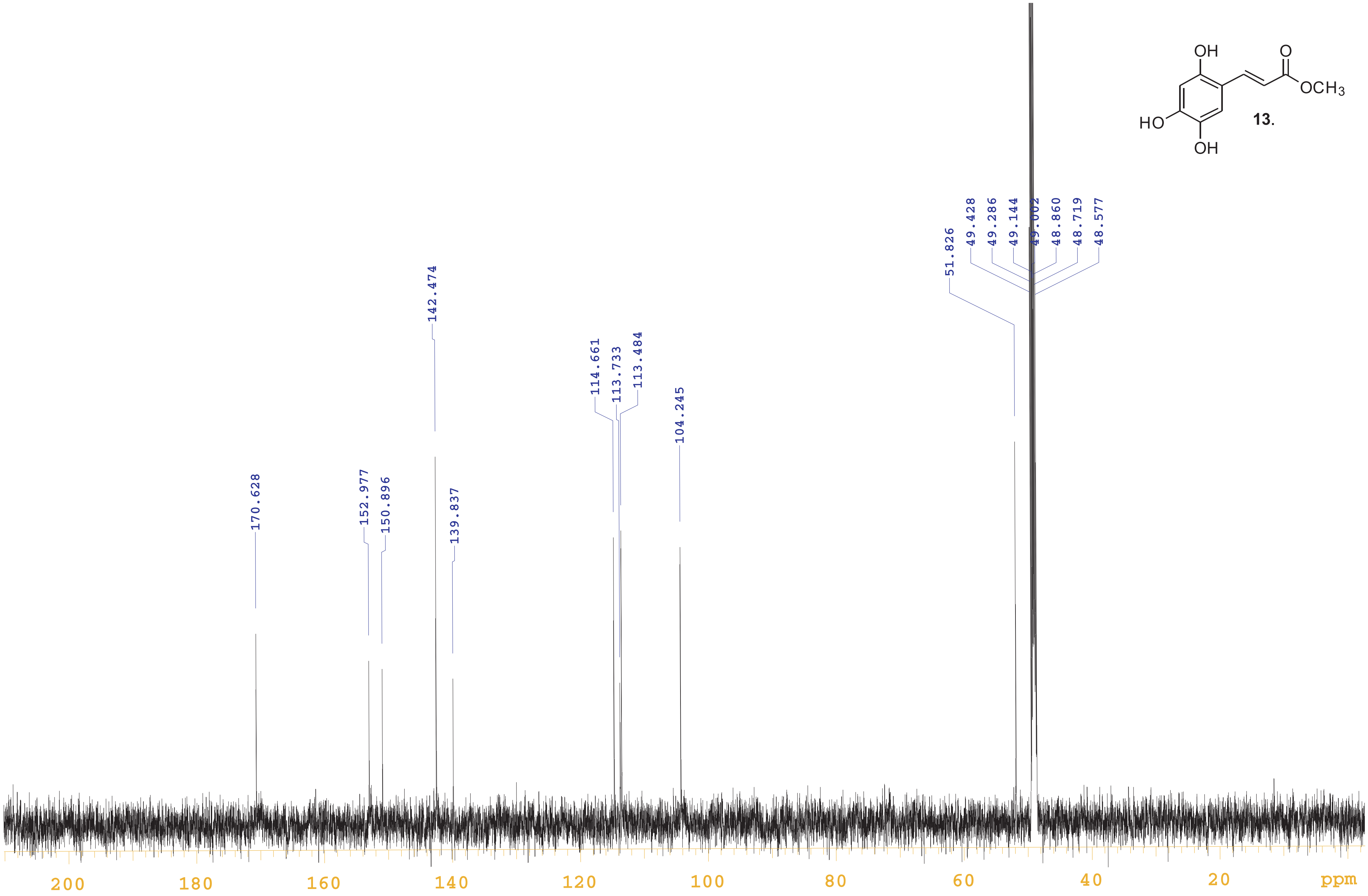

OH
O
OCH3
HO
OH
13.
170.628
152.977
150.896
142.474
139.837
114.661
113.733
113.484
104.245
51.826
49.428
49.286
49.144
49.002
48.860
48.719
48.577
200
180
160
140
120
100
80
60
40
20
ppm

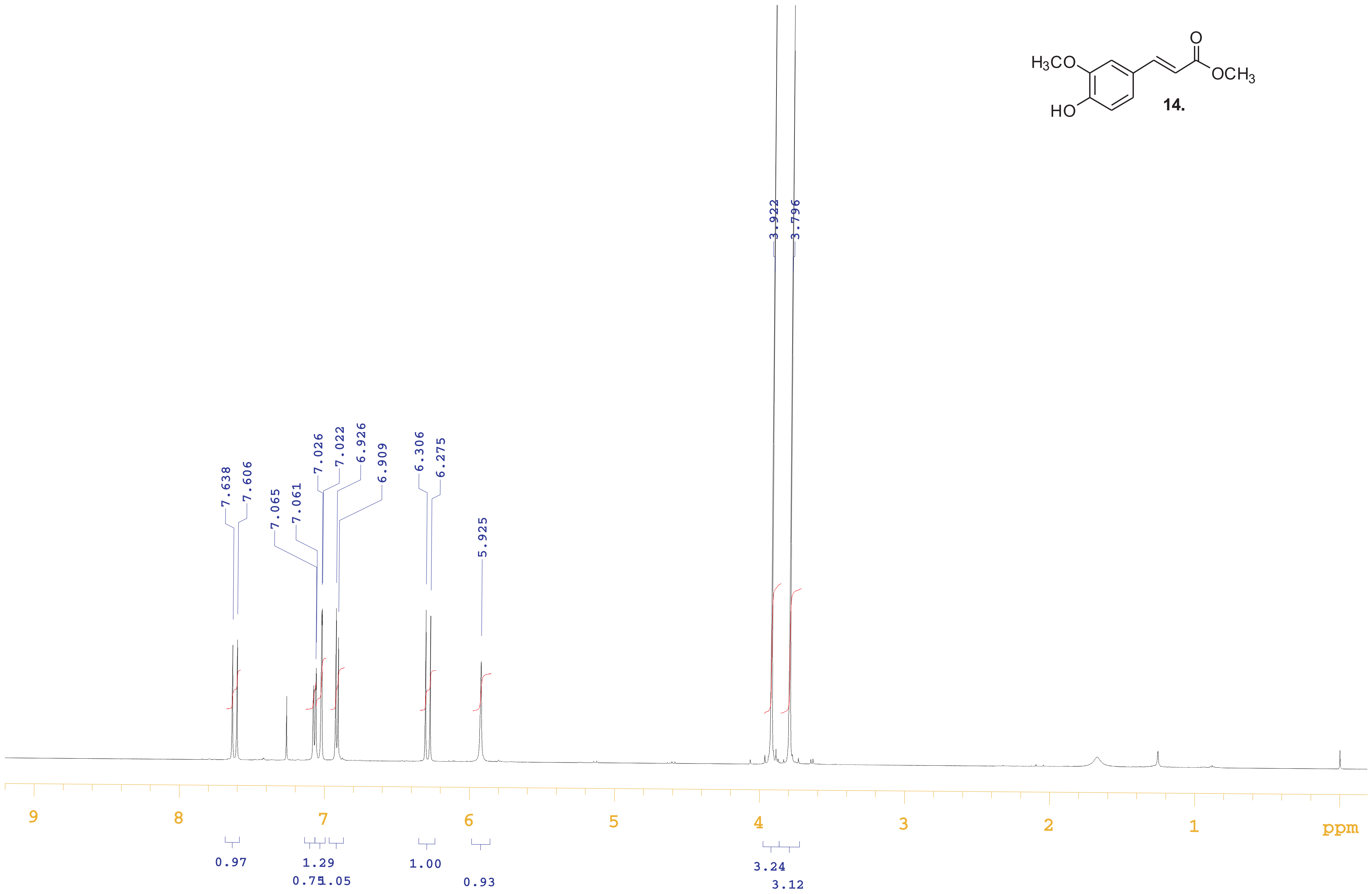

H3CO
HO
O
OCH3
14.
7.638
7.606
7.065
7.061
7.026
7.022
6.926
6.909
6.306
6.275
5.925
3.922
3.796
9
8
7
6
5
4
3
2
1
ppm
0.97
1.29
0.75
1.05
1.00
0.93
3.24
3.12

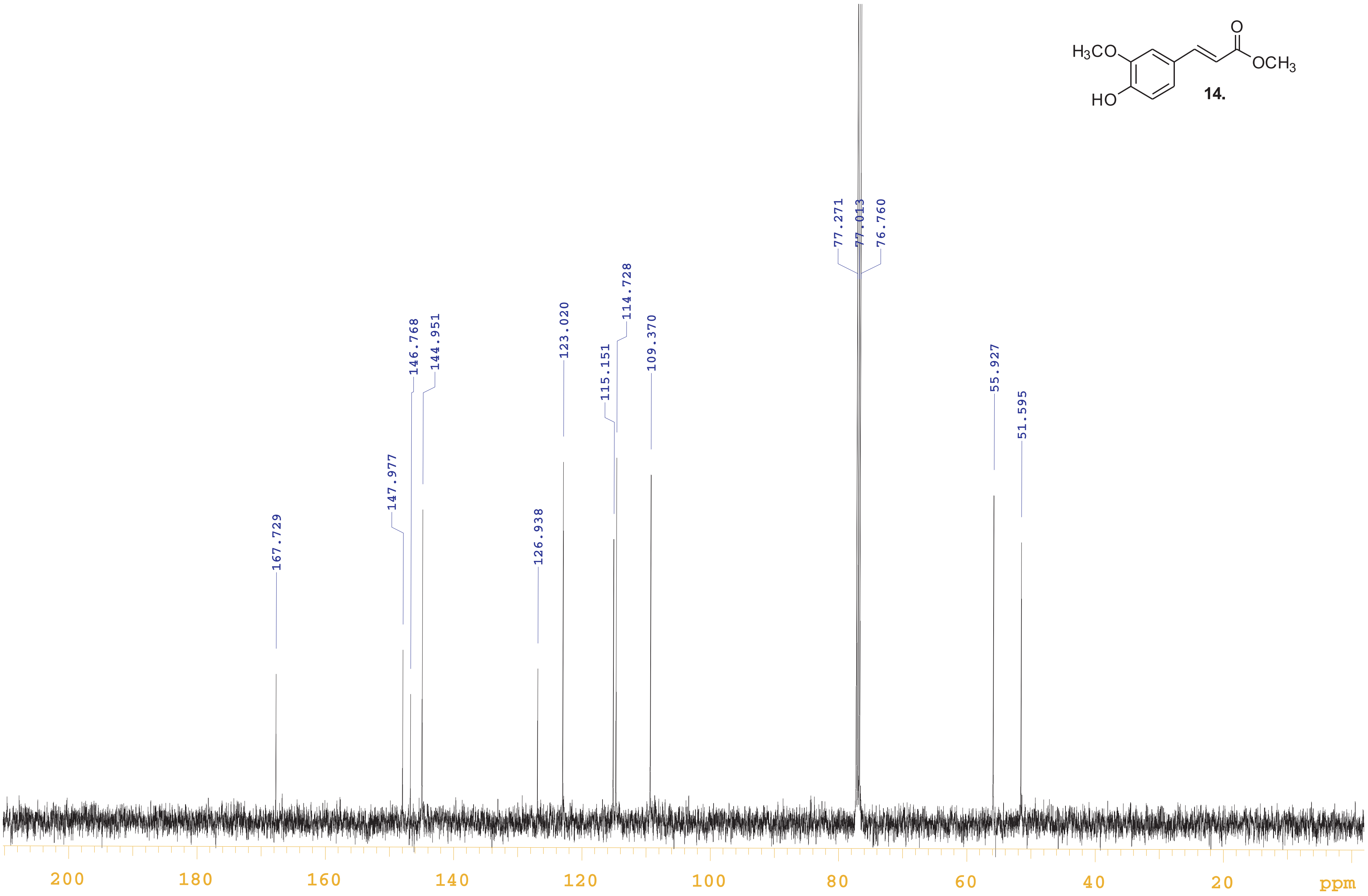
H3CO
HO
O
OCH3
14.
167.729
147.977
146.768
144.951
126.938
123.020
115.151
114.728
109.370
77.271
77.013
76.760
55.927
51.595
200
180
160
140
120
100
80
60
40
20
ppm

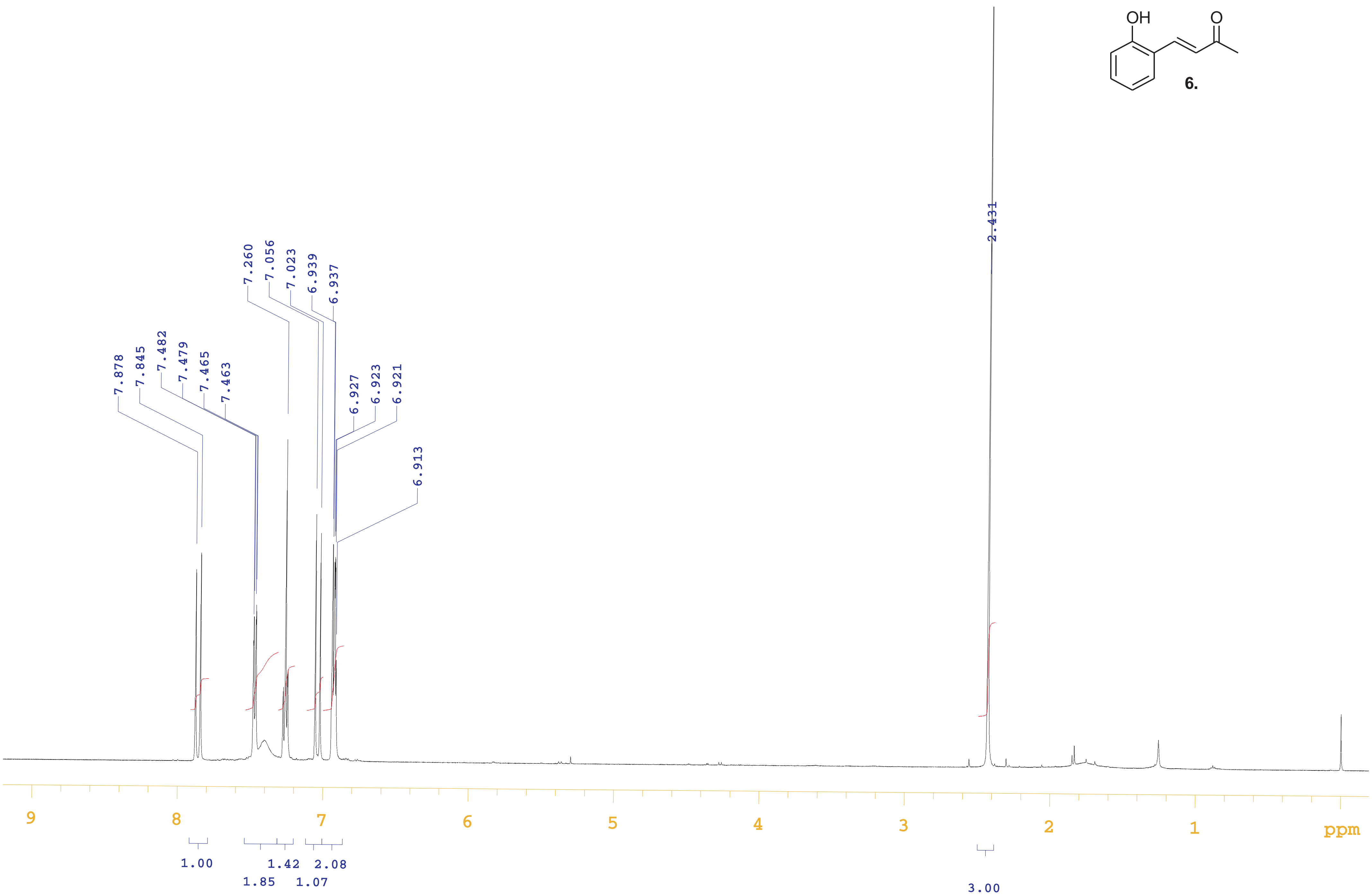

OH
O
6.
7.878
7.845
7.482
7.479
7.465
7.463
7.260
7.056
7.023
6.939
6.937
6.927
6.923
6.921
6.913
2.431
9
8
7
6
5
4
3
2
1
ppm
1.00
1.85
1.42
1.07
2.08
3.00

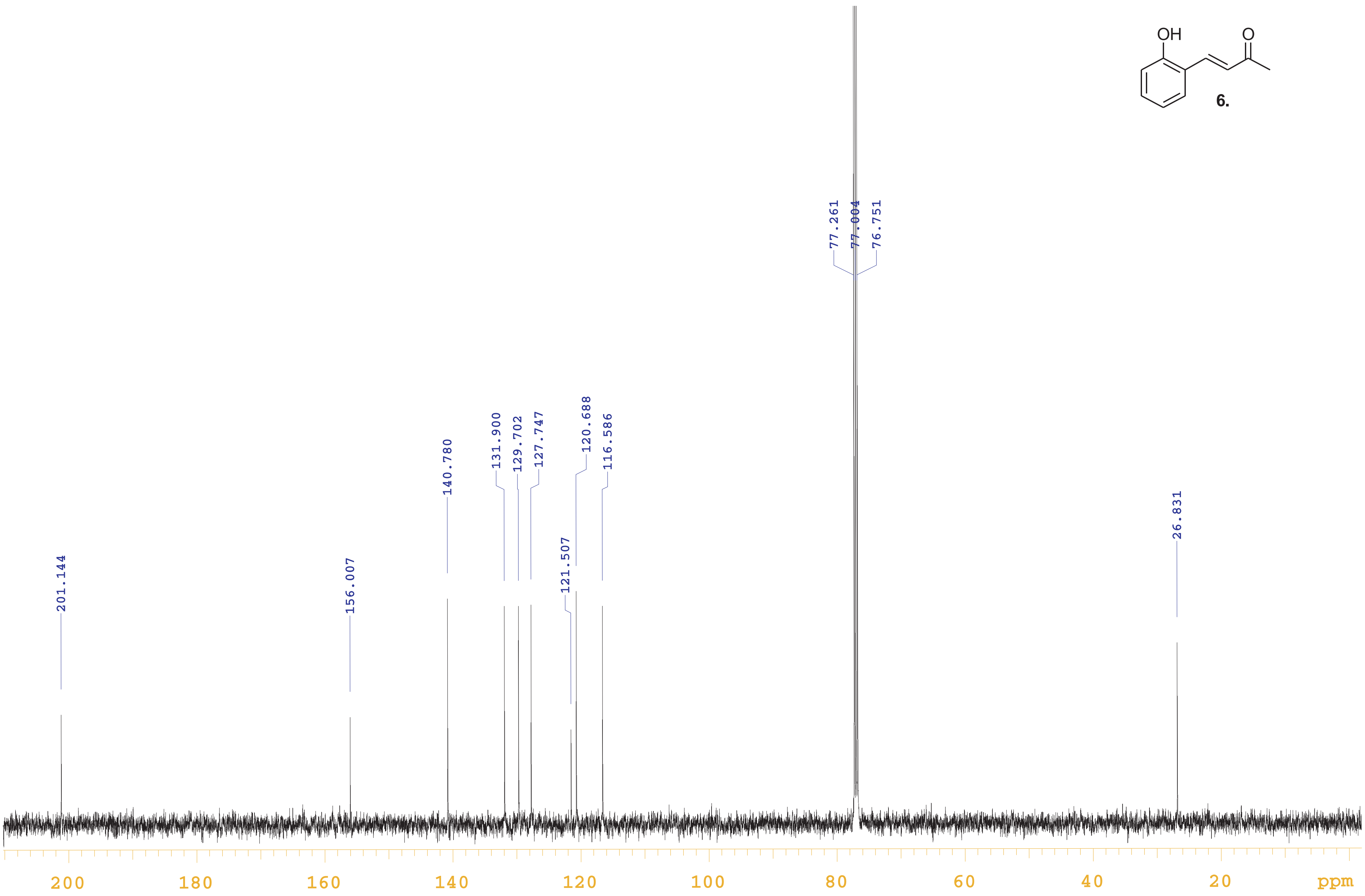
OH
O
6.
201.144
156.007
140.780
131.900
129.702
127.747
121.507
120.688
116.586
77.261
77.004
76.751
26.831
200
180
160
140
120
100
80
60
40
20
ppm

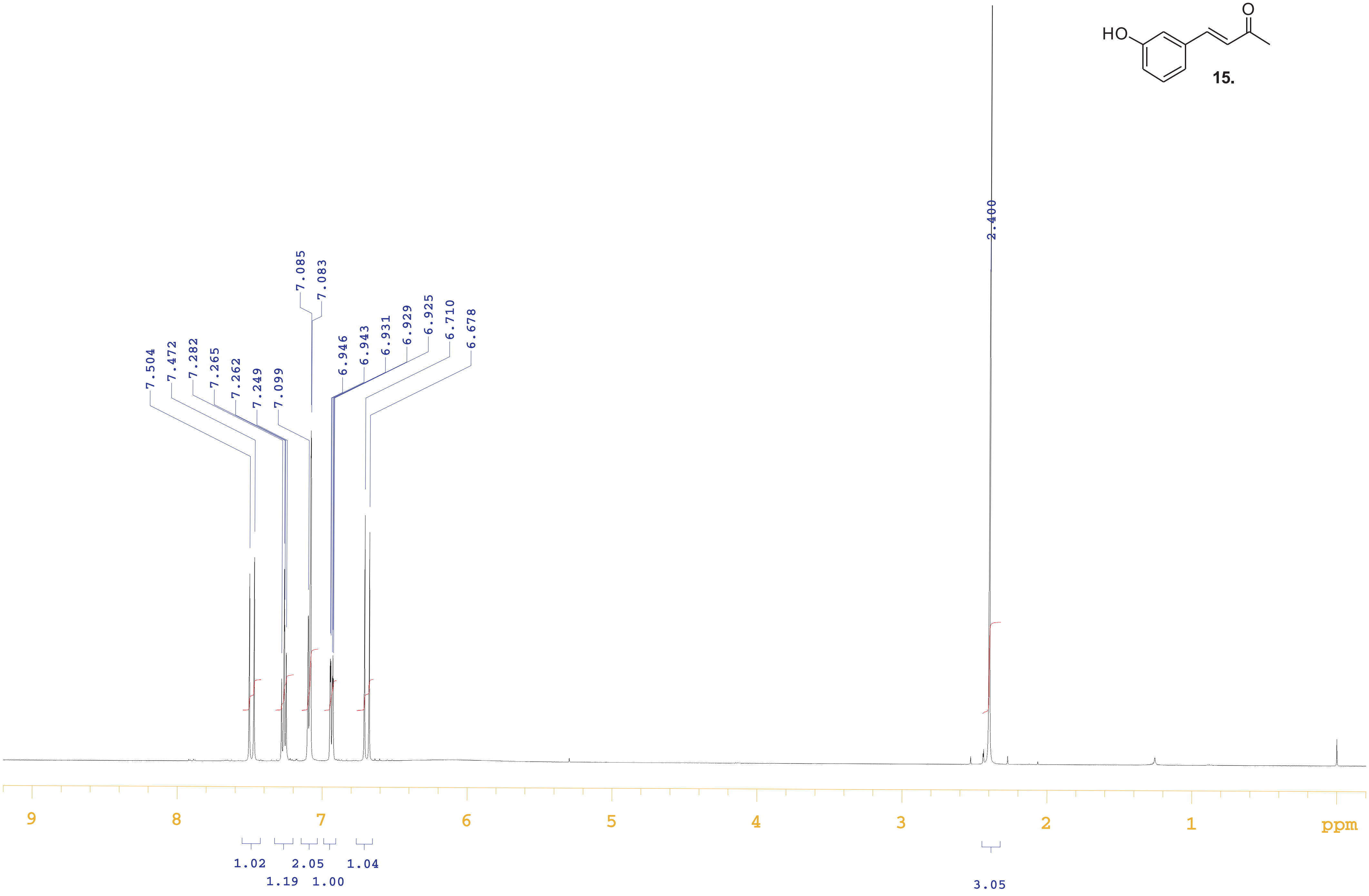
HO
O
15.
7.504
7.472
7.282
7.265
7.262
7.249
7.099
7.085
7.083
6.946
6.943
6.931
6.929
6.925
6.710
6.678
2.400
9
8
7
6
5
4
3
2
1
ppm
1.02
1.19
2.05
1.00
1.04
3.05

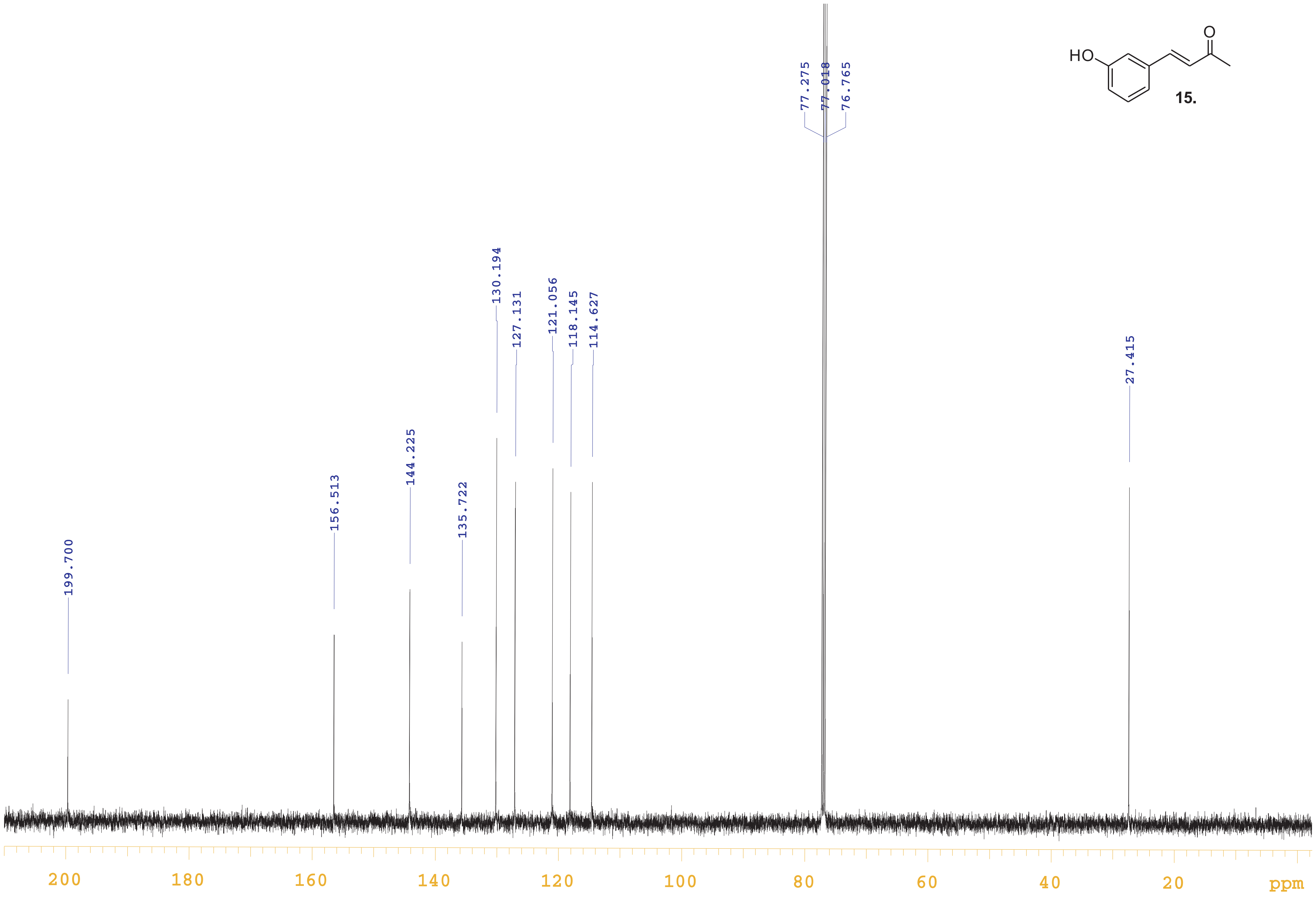
199.700
156.513
144.225
135.722
130.194
127.131
121.056
118.145
114.627
77.275
77.018
76.765
27.415
200
180
160
140
120
100
80
60
40
20
ppm
HO
O
15.

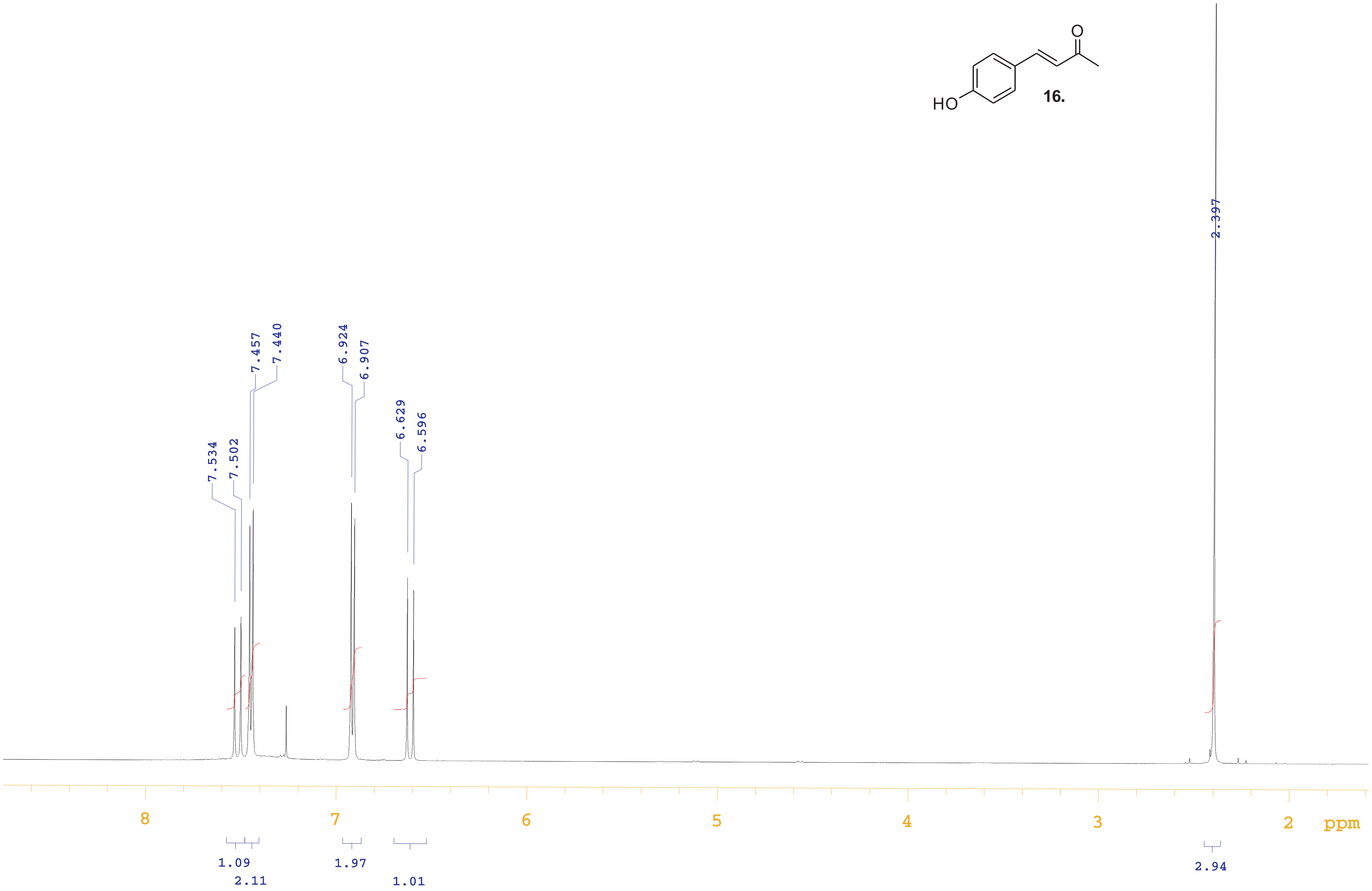
HO
O
16.
7.534
7.502
7.457
7.440
6.924
6.907
6.629
6.596
2.397
8
7
6
5
4
3
2
ppm
1.09
2.11
1.97
1.01
2.94

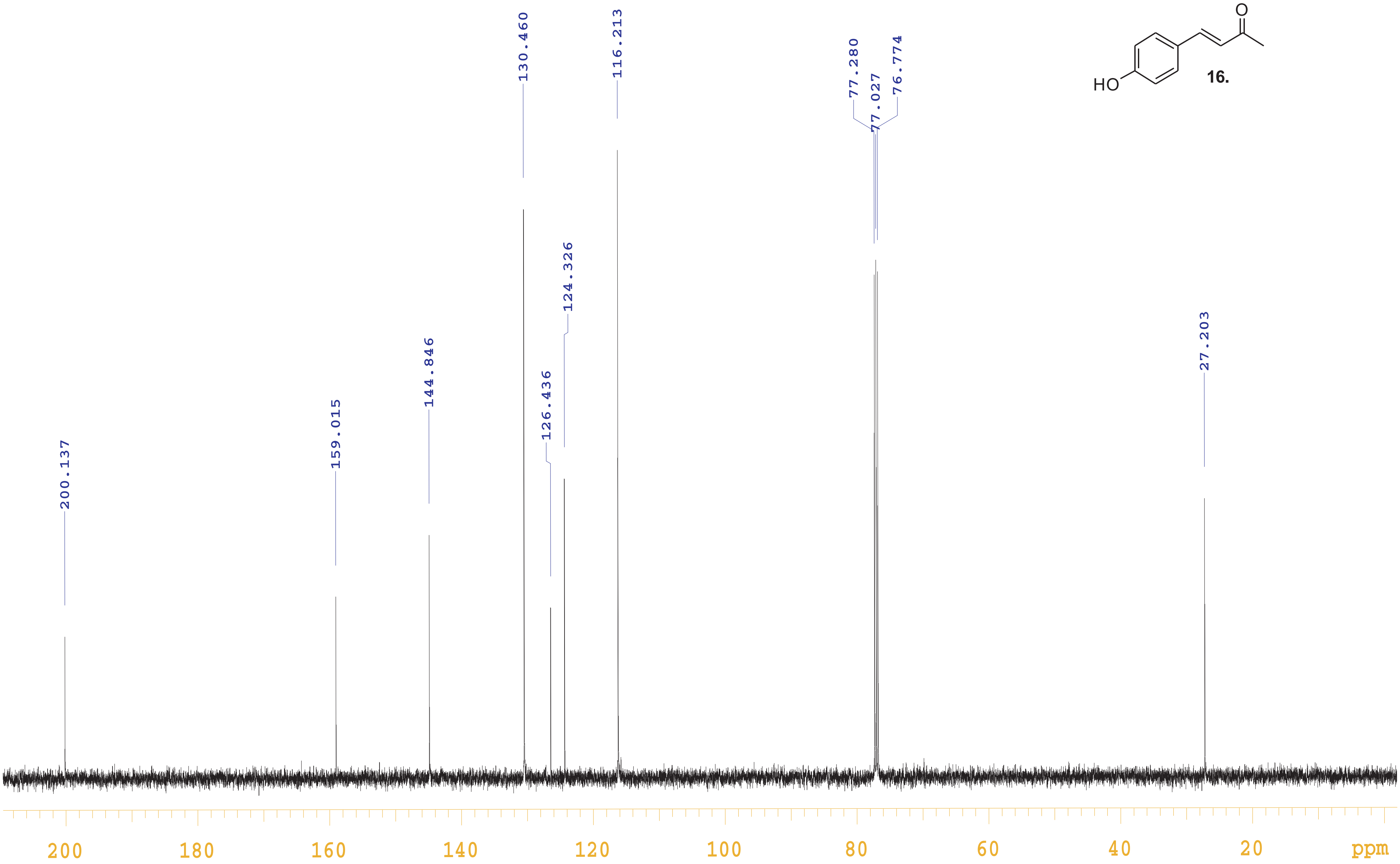

200.137
159.015
144.846
130.460
126.436
124.326
116.213
77.280
77.027
76.774
27.203
HO
O
16.
200
180
160
140
120
100
80
60
40
20
ppm

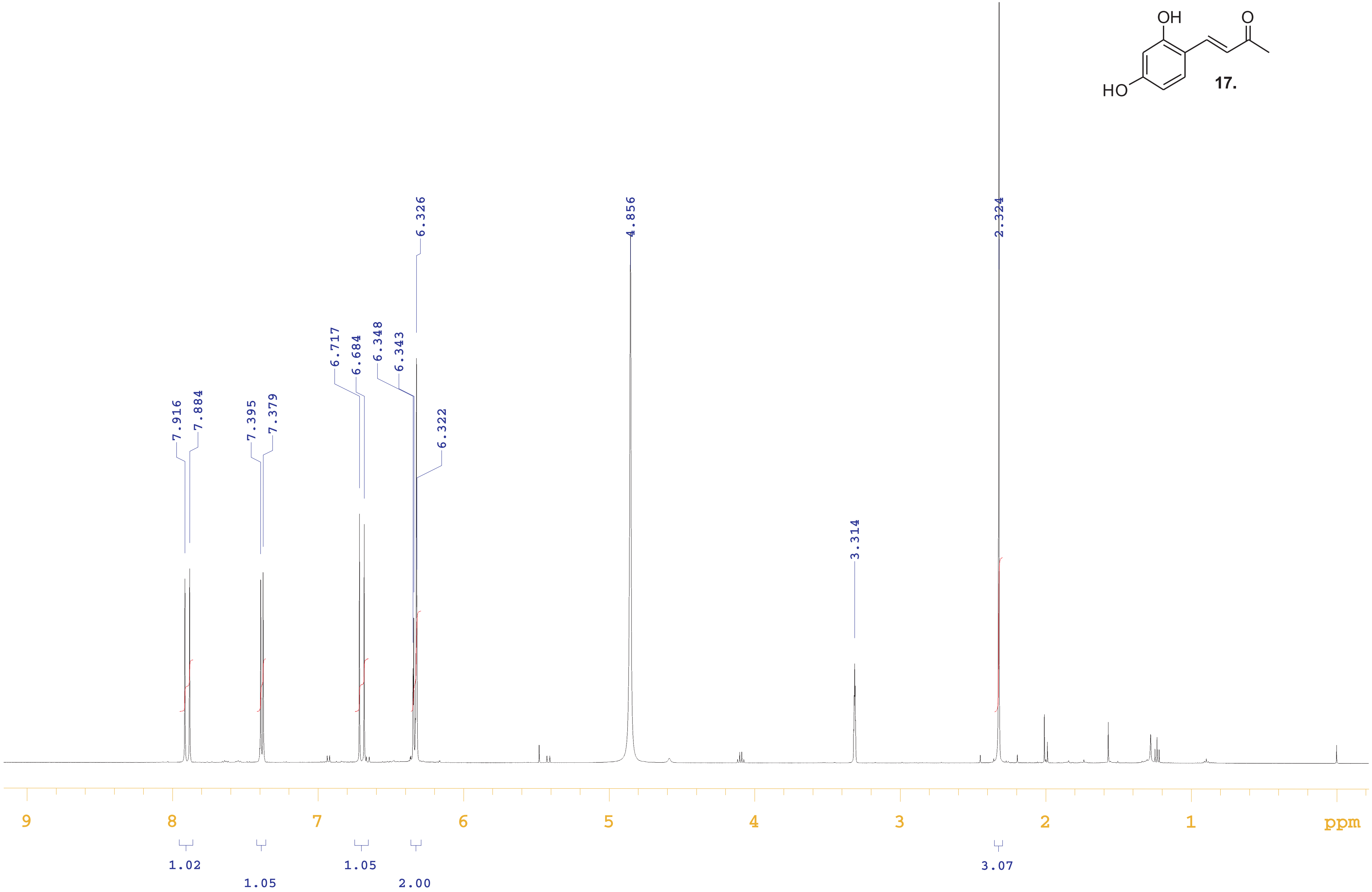

OH
O
HO
17.
7.916
7.884
7.395
7.379
6.717
6.684
6.348
6.343
6.326
6.322
4.856
3.314
2.324
9
8
7
6
5
4
3
2
1
ppm
1.02
1.05
1.05
2.00
3.07

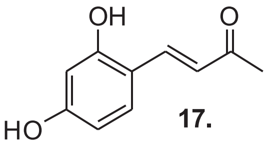
OH
O
HO

17.

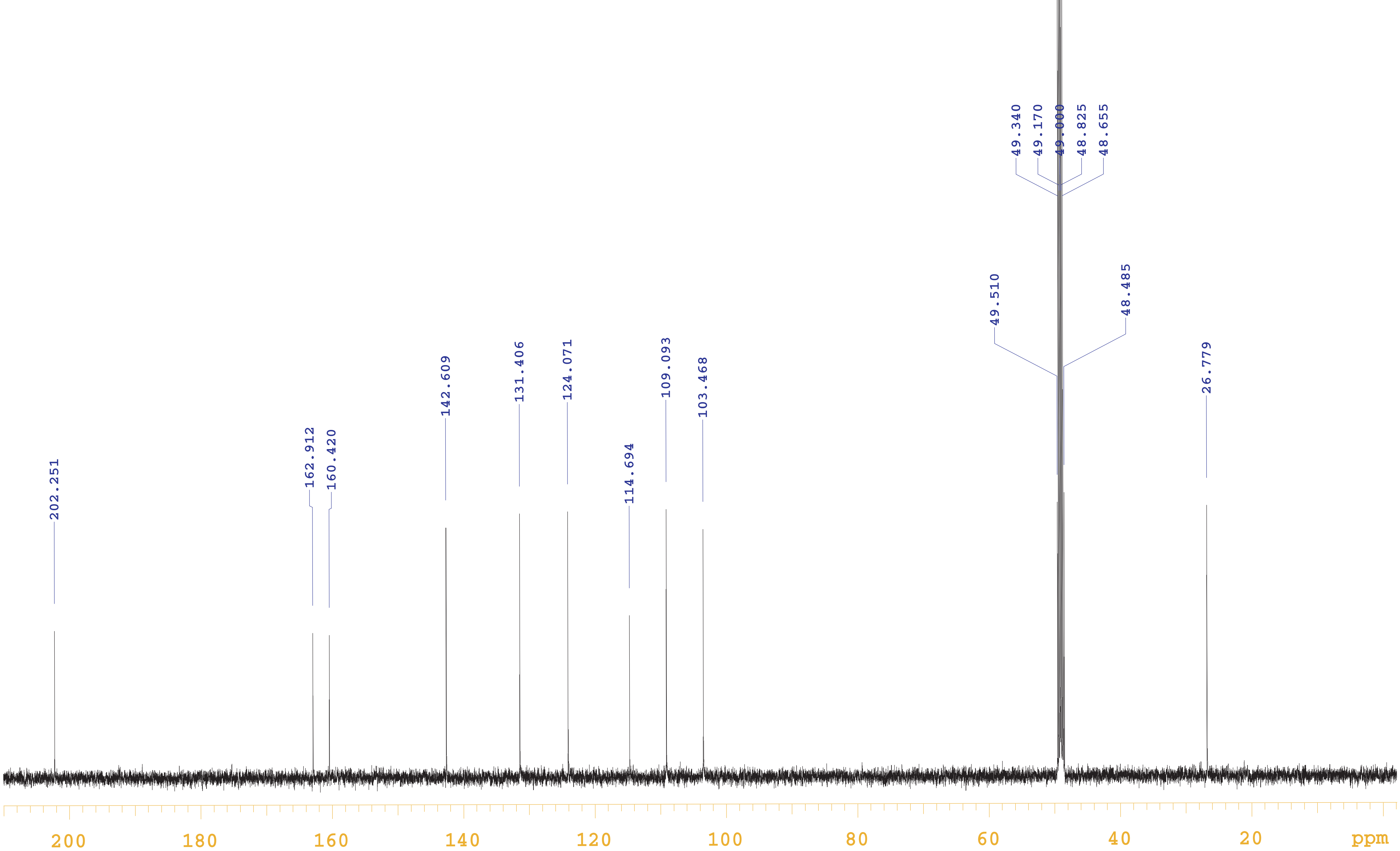
202.251
162.912
160.420
142.609
131.406
124.071
114.694
109.093
103.468
49.510
49.340
49.170
49.000
48.825
48.655
48.485
26.779
200
180
160
140
120
100
80
60
40
20
ppm

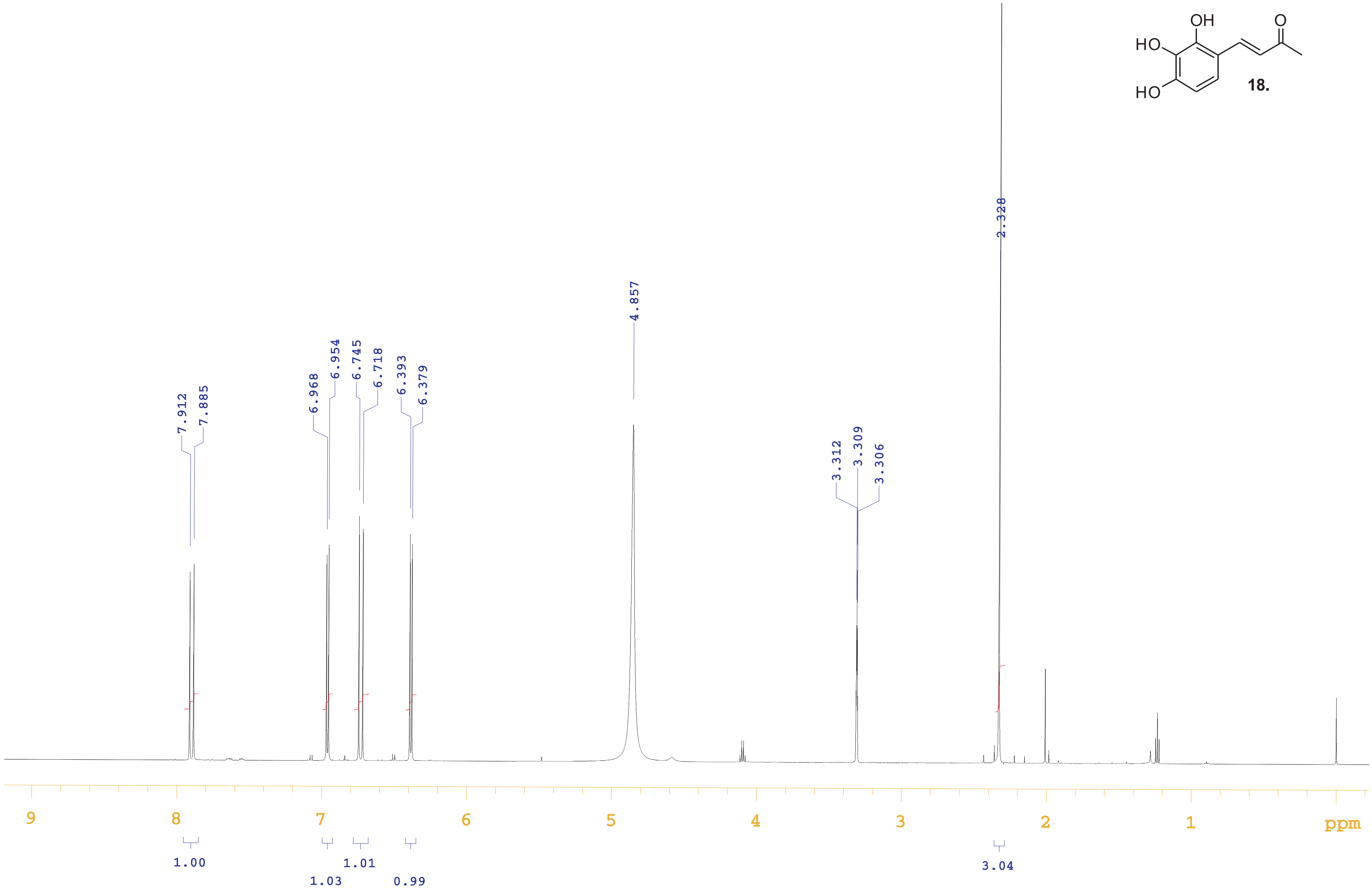

OH
O
HO
HO
18.
7.912
7.885
6.968
6.954
6.745
6.718
6.393
6.379
4.857
3.312
3.309
3.306
2.328
9
8
7
6
5
4
3
2
1
ppm
1.00
1.03
1.01
0.99
3.04

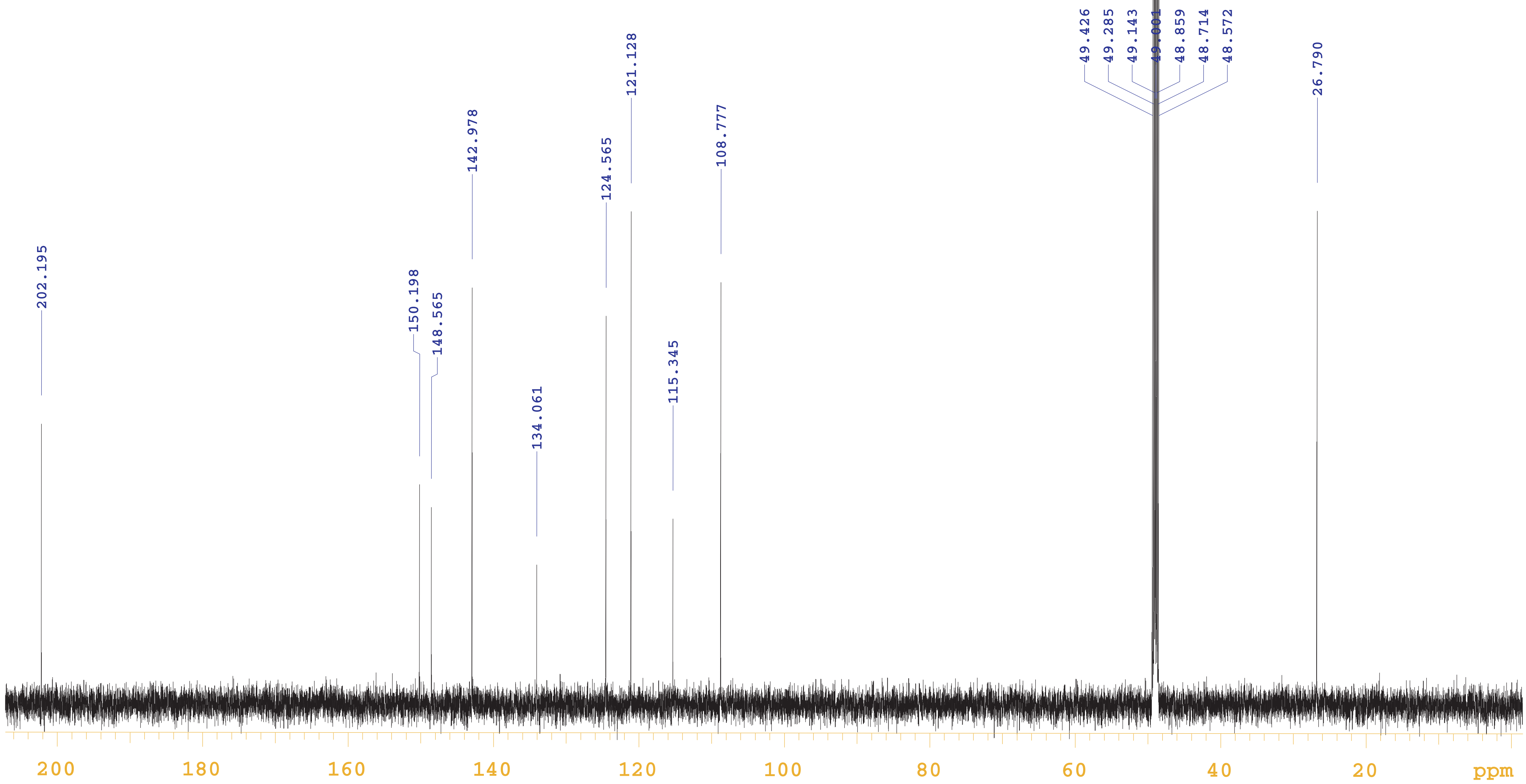
202.195
150.198
148.565
142.978
134.061
124.565
121.128
115.345
108.777
49.426
49.285
49.143
49.001
48.859
48.714
48.572
26.790
200
180
160
140
120
100
80
60
40
20
ppm

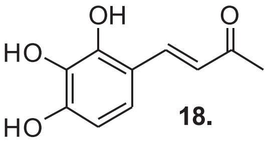
OH
O
HO
HO

18.

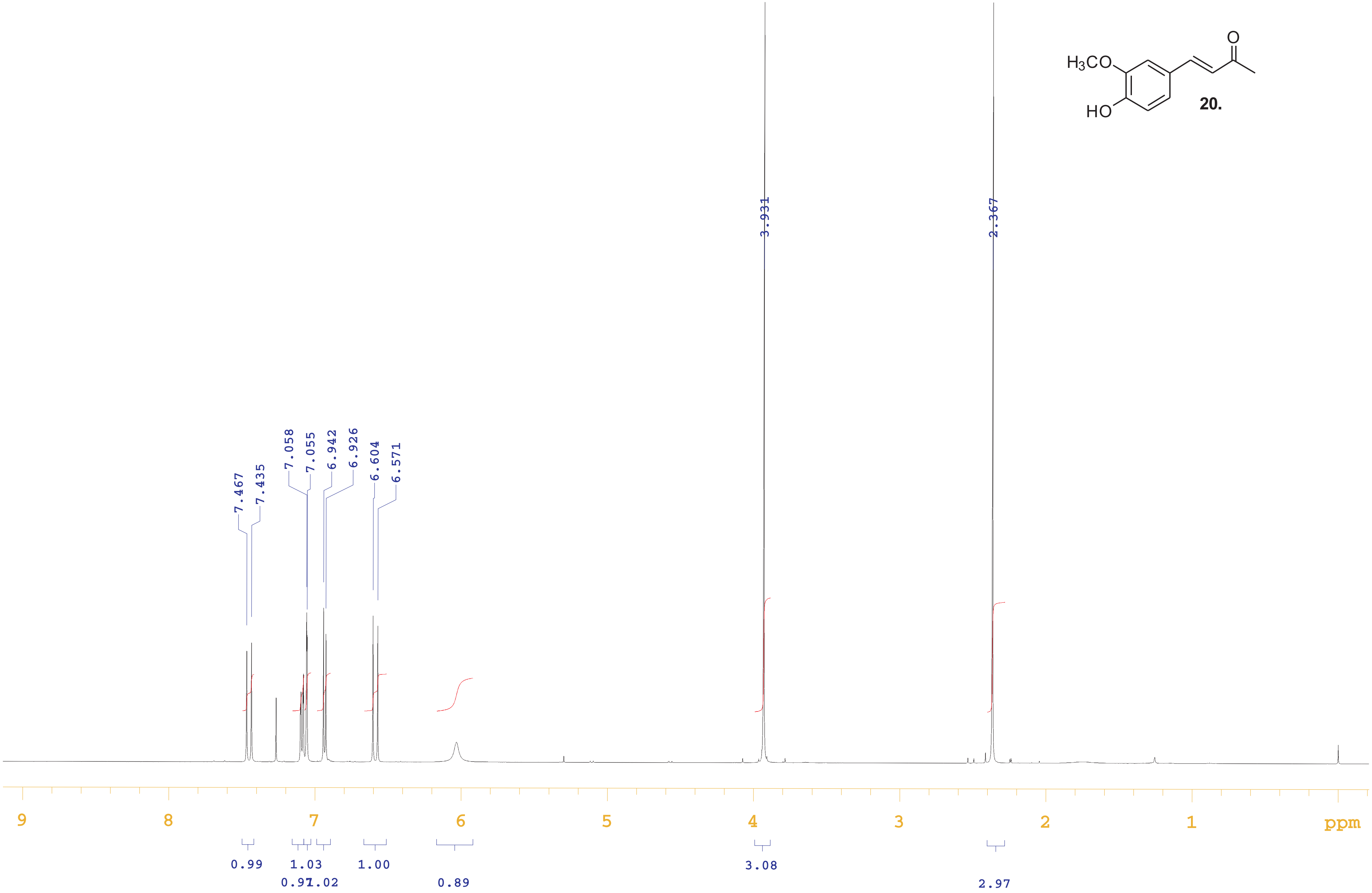
H3CO
HO
O
20.
7.467
7.435
7.058
7.055
6.942
6.926
6.604
6.571
3.931
2.367
9
8
7
6
5
4
3
2
1
ppm
0.99
1.03
0.97
1.02
1.00
0.89
3.08
2.97

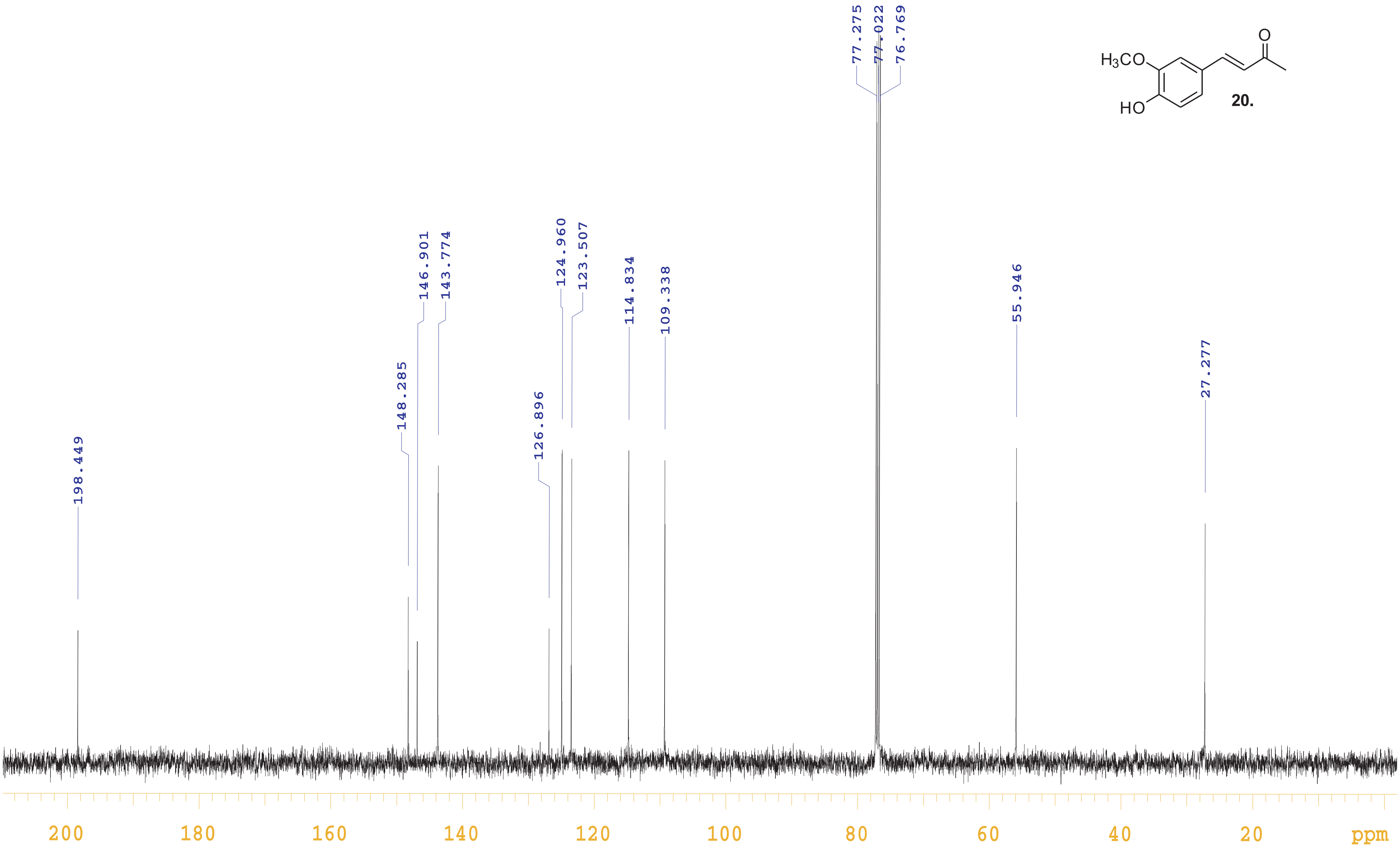
198.449
148.285
146.901
143.774
126.896
124.960
123.507
114.834
109.338
77.275
77.022
76.769
55.946
27.277
200
180
160
140
120
100
80
60
40
20
ppm
H3CO
HO
O
20.
